# The research on the identification, taxonomy, and comparative genomics analysis of nine *Bacillus velezensis* strains significantly contributes to microbiology, genetics, bioinformatics, and biotechnology

**DOI:** 10.3389/fmicb.2025.1544934

**Published:** 2025-03-19

**Authors:** Eduarda Guimarães Sousa, Gabriela Munis Campos, Marcus Vinícius Canário Viana, Gabriel Camargos Gomes, Diego Lucas Neres Rodrigues, Flavia Figueira Aburjaile, Belchiolina Beatriz Fonseca, Max Roberto Batista de Araújo, Mateus Matiuzzi da Costa, Eric Guedon, Bertram Brenig, Siomar Soares, Vasco Azevedo

**Affiliations:** ^1^Cellular and Molecular Genetics Laboratory, Department of General Biology, Institute of Biological Sciences, Federal University of Minas Gerais, Belo Horizonte, Minas Gerais, Brazil; ^2^Integrative Bioinformatics Laboratory, Veterinary School, Federal University of Minas Gerais, Belo Horizonte, Minas Gerais, Brazil; ^3^Postgraduate Program in Veterinary Sciences and Postgraduate Program in Genetics and Biochemistry at Federal University of Uberlandia, Uberlândia, Minas Gerais, Brazil; ^4^Operational Technical Nucleus, Microbiology, Hermes Pardini Institute (Fleury Group), Vespasiano, Minas Gerais, Brazil; ^5^Materials Science Research Institute, Federal University of the São Francisco Valley, Juazeiro, Bahia, Brazil; ^6^STLO, INRA, Agrocampus Ouest, Rennes, France; ^7^Institute of Veterinary Medicine, University of Göttingen, Göttingen, Germany; ^8^Laboratory of Bioinformatics, Department of Microbiology, Immunology and Parasitology, Institute of Biological and Natural Sciences, Federal University of Triângulo Mineiro, Uberaba, Minas Gerais, Brazil

**Keywords:** identification, characterization, sequencing, *Bacillus velezensis*, genomic

## Abstract

**Introduction:**

Next-generation sequencing (NGS) has played a pivotal role in the advancement of taxonomics, allowing for the accurate identification, differentiation, and reclassification of several bacteria species. *Bacillus velezensis* is a Gram-positive, facultatively aerobic, spore-forming bacterium known for its antimicrobial and antifungal properties. Strains of this species are highly relevant in agriculture, biotechnology, the food industry, and biomedicine.

**Methods:**

In this study, we characterized the genomes of nine *Bacillus* strains isolated from soil in the state of Bahia (Brazil) using NGS with Illumina platform. Identification was performed by Average Nucleotide Identity (ANI) and digital DNA-DNA hybridization (dDDH) analyses, which revealed a match between the genomic information of the isolates and *B. velezensis* NRRL B-41580, with a variation of 89.3% to 91.8% by dDDH in TYGS and 95% to 98.04% by ANI in GTDBtk.

**Results and discussion:**

Two strains, BAC144 and BAC1273, exhibited high similarity to *B. amyloliquefaciens subsp. plantarum* FZB42. However, the latter strain was subsequently reclassified as *B. velezensis*. The division pattern observed during identification was confirmed in the phylogenomic analysis, where BAC144 and BAC1273 clustered with *Bacillus amyloliquefaciens subsp. plantarum*, while the other strains clustered with *B. velezensis* NRRL B-41580, forming a clade with high genetic similarity, with a bootstrap value of 100%. Furthermore, a synteny analysis demonstrated greater conservation among the strains from this study compared to the reference strain, with the formation of distinct collinear groups. The pangenome analysis revealed an open pangenome, highlighting the genetic diversity within the species. Based on this analysis, a functional annotation was performed to compare exclusive gene repertoires across groups, uncovering distinct adaptations and functional profiles. The identification of bacterial strains belonging to this species is of great importance due to their high applicability. The strains identified in this study underscore the need for more robust taxonomic technologies to accurately classify prokaryotes, which are subject to constant evolutionary changes, requiring the reclassification of several species within the genus Bacillus, many of which are heterotypic synonyms of *B. velezensis* like *Bacillus oryzicola, B. amyloliquefaciens subsp. plantarum* and *Bacillus methylotrophicus*.

## 1 Introduction

The genus *Bacillus* contains species of Gram-positive, rod-shaped, spore-forming, and facultative aerobic bacteria that occur in diverse natural and human-created environments, with environmental, biotechnological, and medical relevance. Some strains have agriculture, bioremediation, and pharmaceutical production applications, while others are human pathogens (Logan and Vos, 2015). After comparative phylogenomic analysis, most of the species were reclassified to other genera, with the remaining ones classified as “*Bacillus subtilis* group” (27 species), or “*Bacillus cereus* group” (19 species). The taxonomy of the *B. subtilis* group underwent various changes, including novel species and many reclassifications, and much work has to be done to benefit the research and the applications (Xu and Kovács, 2024).

*Bacillus velezensis* has agricultural, biotechnological, and environmental applications, due to the promotion of plant growth, inhibition of plant pathogens, probiotic effects in animal feed, production of biopolymers, antimicrobials, anticancer drugs, biosurfactants, and degradation of agroindustrial byproducts (Adeniji et al., 2019; Khalid et al., 2021; Keshmirshekan et al., 2024). The correct identification of *B. velezensis* strains is relevant due to their potential applications.

Taxono-genomics incorporates genome sequencing in taxonomic studies, offering reliable and reproducible data (Ramasamy et al., 2014). The taxonomic identification of bacteria from DNA sequences can be achieved using one of a few loci, such as 16S rRNA, *rpoB* (Christensen and Olsen, 2018), many loci, such as in the method Ribosomal Multilocus Sequence Typing (rMLST) (Jolley et al., 2012), or the whole genome for a more excellent resolution, in methods such as Average Nucleotide Identity (ANI), digital DNA–DNA hybridization (dDDH) (Christensen and Olsen, 2018), and Tetra-nucleotide Signature Correlation Index (TETRA) (Richter and Rosselló-Móra, 2009). Taxono-genomics have been used to describe new species (Ramasamy et al., 2014), and solve taxonomic classifications (Xu and Kovács, 2024).

The methods using whole genome, perform a pairwise comparison of the query sequence to a database of reference genomes, followed by a minimum cutoff value to consider them as from the same species, such as ≥95% for ANI (Jain et al., 2018). ANI facilitates taxonomic identification by leveraging the mean identity of all orthologous genes across a minimum of two genomes. This calculation enables the delineation of lineages within a single species. dDDH simulates traditional DNA–DNA hybridization techniques in silico, providing a similarity score between two genomes. A threshold of >70% similarity indicates that the compared genomes belong to the same species (Meier-Kolthoff et al., 2014). For TETRA a > 0.99 correlation coefficient (z-score) (Richter and Rosselló-Móra, 2009). Difference in G + C content of not greater than 1% is also expected within genomes from the same species (Meier-Kolthoff et al., 2014).

Previous studies have reported the importance of *B. velezensis* strains as potential probiotics, mainly applied in aquaculture and agriculture (Khalid et al., 2021; Dong et al., 2022). However, it has also been applied to humans, as shown in the work of Brutscher et al. (2024), where strain BV379 was evaluated for biosafety as a potential candidate probiotic for oral use. One of the most beneficial effects of *B. velezensis* for these applications is the production of bioactive compounds such as surfactin, bacilysin and fengycin, which are associated with antagonistic, biosurfactant, antioxidant and anti-inflammatory activities (Han X. et al., 2021; Barale et al., 2022; Medeot et al., 2023).

Given the high biotechnological applicability of this species, this work identified and characterized the genomes of nine strains isolated from soil in Bahia, Brazil, where the discovery of new genetic profiles will reinforce the importance of studies on its diversity and evolution, showing its value for future industrial and therapeutic applications, and the need to characterize strains for biological control or use as probiotics.

## 2 Materials and methods

### 2.1 Isolated organisms and identification by MALDI-TOF MS of cultivable bacteria

Nine *Bacillus* strains, BAC39, BAC118, BAC124, BAC137, BAC144, BAC156, BAC207, BAC238, and BAC1273 (Supplementary Table 1), isolated from the soil in Bahia, Brazil, were used. They were cultivated in Lysogeny Broth (LB) supplemented with 0.1% Tween 80 for 24 h at 37°C.

Furthermore, a Flex Control Microflex LT mass spectrometer (Bruker Daltonics) with a 60 Hz nitrogen laser was used to process the spectra of each sample using Matrix-Assisted Laser Desorption/Ionization Time-of-Flight Mass Spectrometry (MALDI-TOF MS). This technique enables rapid bacterial identification by analyzing protein mass spectra. The MALDI Biotyper software, version 3 (Bruker Daltonics), was used to create a Master Spectral Library (MSP) for the strains, utilizing the BioTyper MSP breeding standard. The bacteria were prepared in accordance with the manufacturer’s instructions (Dos Santos et al., 2022).

DNA extraction was performed through the Wizard^®^ Genomic DNA Purification Kit (Promega), following the instructions recommended by the manufacturer. Next-generation sequencing was performed using the Hi-Seq 2,500 platform (2x150bp) (Illumina^®^, United States), with the ThruPLEX DNA-Seq Kit (Takara) used for paired-end library construction.

### 2.2 Genomic data from public databases

Two different datasets were used for phylogenomic analyses. One dataset contains all *Bacillus* genomes from RefSeq, deposited in the National Center for Biotechnology Information (NCBI) database (accessed on 10 May 2024), and also present in the Genome Taxonomy Database (GTDB), as listed in Supplementary Table 2, with *Pseudomonas aeruginosa* DSM 50071^T^ as an outgroup (Gupta et al., 2023), the *B. velezensis NRRL B-41580* and *Bacillus amyloliquefaciens subsp. plantarum FZB42* strains were also included in the dataset, identified by analyzing their Average Nucleotide Identity (ANI), which showed a similarity of over 95% to the samples in the collection, organisms belonging to the same species or closely related represent a cutoff value of ≥95% (Jain et al., 2018).

For the second dataset, strains that showed alignment with the isolated samples predicted by the Type Strain Genome Server^1^ were used for phylogenomic analyses, as listed in Table 1 and Supplementary Data 1. All genomes were downloaded from the RefSeq NCBI (National Center for Biotechnology Information) databases.

**Table 1:** Genome information for the dataset based on TYGS analysis.

GenBank access	Organism	Genome size (Mb)	GC content (%)	Assembly level	Geographic location of isolation
GCF_001461825.1	*Bacillus velezensis* NRRL_B-41580	4.0	46.5	Contig	Spain
GCF_000015785.2	*Bacillus amyloliquefaciens subs. Plantarum* FZB42	3.9	46.5	Complete	Germany
GCF_001461835.1	*Bacillus oryzicola* KACC 18228 T	3.9	46.5	Contig	South Korea
GCF_000960265.2	*Bacillus methylotrophicus* KACC 13105	3.9	46.5	Contig	South Korea
GCF_000262045.1	*Bacillus siamensis* KCTC 13613	3.8	46.5	Contig	South Korea
GCF_000966575.1	*Bacillus vanillea* XY18	3.7	46.5	Contig	China
GCF_000196735.1	*Bacillus amyloliquefaciens* DSM7	4.0	46.0	Complete	Germany
GCF_001584325	*Bacillus nakamurai* NRRL B-41091	3.7	45.5	Contig	Argentina
GCF_900445435.1	*Bacillus tequilensis* NCTC13306	3.7	44.0	Contig	United Kingdom
GCF_000186085.1	*Bacillus subtilis* NCIB 3610	4.3	43.5	Chromosome	United States
GCF_018390475.2	*Bacillus cabrialesii subsp. tritici* TSO2	4.3	44.0	Contig	Mexico
GCF_000344745.1	*Bacillus subtilis* ATCC 6051	4.2	43.5	Complete	Germany
GCF_011745685.2	*Bacillus rugosus* SPB7	4.5	43.0	Contig	India
GCF_000507105.1	*Bacillus mojavensis* KCTC 3706	3.9	43.5	Contig	South Korea
GCF_000245315.1	*Bacillus vallismortis* DV1-F-3	3.9	44.0	Scaffold	United States
GCF_020223575.1	*Bacillus mexicanus* FSQ1	3.6	43.0	Contig	Mexico

### 2.3 Assembly

The trimming and quality of the sequencing were analyzed using the Fastp v0.23.4 tool,^2^ an Ultrafast one-pass FASTQ data preprocessing, to assemble and annotate the genomes a posteriori, as indicated in the reports generated by the sequencing software (Babraham Bioinformatics – FastQC A Quality Control tool for High Throughput Sequence Data, n.d.; Chen, 2023). The *de novo* assembly software for prokaryotes, Unicycler v0.5.1 (Wick et al., 2017), was used to assemble the sequenced isolates. The Quast software^3^ (Gurevich et al., 2013) was used to evaluate the quality of the assembly.

### 2.4 Quality and contamination

The isolated, assembled, and annotated genomes were analyzed to assess their quality and identify potential errors, including contamination, completeness, and genome integrity. This was performed using the CheckM2 v1.0.2 software (Chklovski et al., 2023),^4^ which use universal machine learning models that performed analyses based on the amount of GC content. The presence of chimeric contigs was analyzed using GUNC v1.0.6 (Orakov et al., 2021).^5^

### 2.5 Annotation

The annotation was performed using the Prokka software, designed for rapid prokaryotic genome annotation^6^ (Seemann, 2014). Prokka uses a variety of databases to identify the functional elements (features) of bacterial and archaeal genomes, including coding DNA sequences (CDSs), tRNAs, rRNAs, and protein functional annotations. The output is compatible with files that are suitable for use in subsequent analyses.

### 2.6 Genomic approaches for taxonomic, pangenome and phylogenetic insights

The taxonomic identification of the strains was determined using two distinct approaches. The first approach was the digital DNA–DNA Hybridization (dDDH) analysis implemented in TYGS^7^ (Meier-Kolthoff et al., 2013; Meier-Kolthoff and Göker, 2019). This method calculates the DNA similarity between the aligned fragments of a query genome to a Type Strain database, with a similarity cutoff above 70% for the same species. As a second approach to identification, ANI was analyzed using the GTDB-Tk v2.4.0,^8^ which performs taxonomic classification based on the Genome Database Taxonomy (GTDB) (Chaumeil et al., 2020). For this study, similarities more significant than 95% were considered to be indicative of a close genetic relationship (Ciufo et al., 2018).

The analogous genomes generated by TYGS were used as a dataset for subsequent analyses, including the heatmap construction to corroborate the identification of each lineage by the pyANI program v3.0 (Pritchard et al., 2019). This program generates the map by calculating the ANI through the MUMmer alignment method, with a 95% similarity threshold for distinct genomes of the same species.

Following the MLST scheme defined in PubMLST, the sequence type (ST) was determined *in silico* using the FastMLST script,^9^ taking into account the alleles of seven housekeeping genes (Guerrero-Araya et al., 2021). In addition, PhyloPhlAn v 3.1.68,^10^ an integrated pipeline for large-scale phylogenetic profiling (Asnicar et al., 2020), was used to construct a tree based on maximum likelihood strategies of the core genome generated by the PPanGGOLiN program v 2.2.0 and bootstrap values with 1,000 replicates. Moreover, a phylogenetic tree based on the 16S rRNA gene was constructed. The sequences were obtained from genomic annotation and the GenBank database at NCBI. The phylogenetic analysis was performed using MEGA version 12, using the maximum likelihood method with bootstrap values calculated from 1,000 replicates (Kumar et al., 2024). The both trees were visualized using iTOL v.6^11^ (Letunic and Bork, 2024).

**Figure 1:**
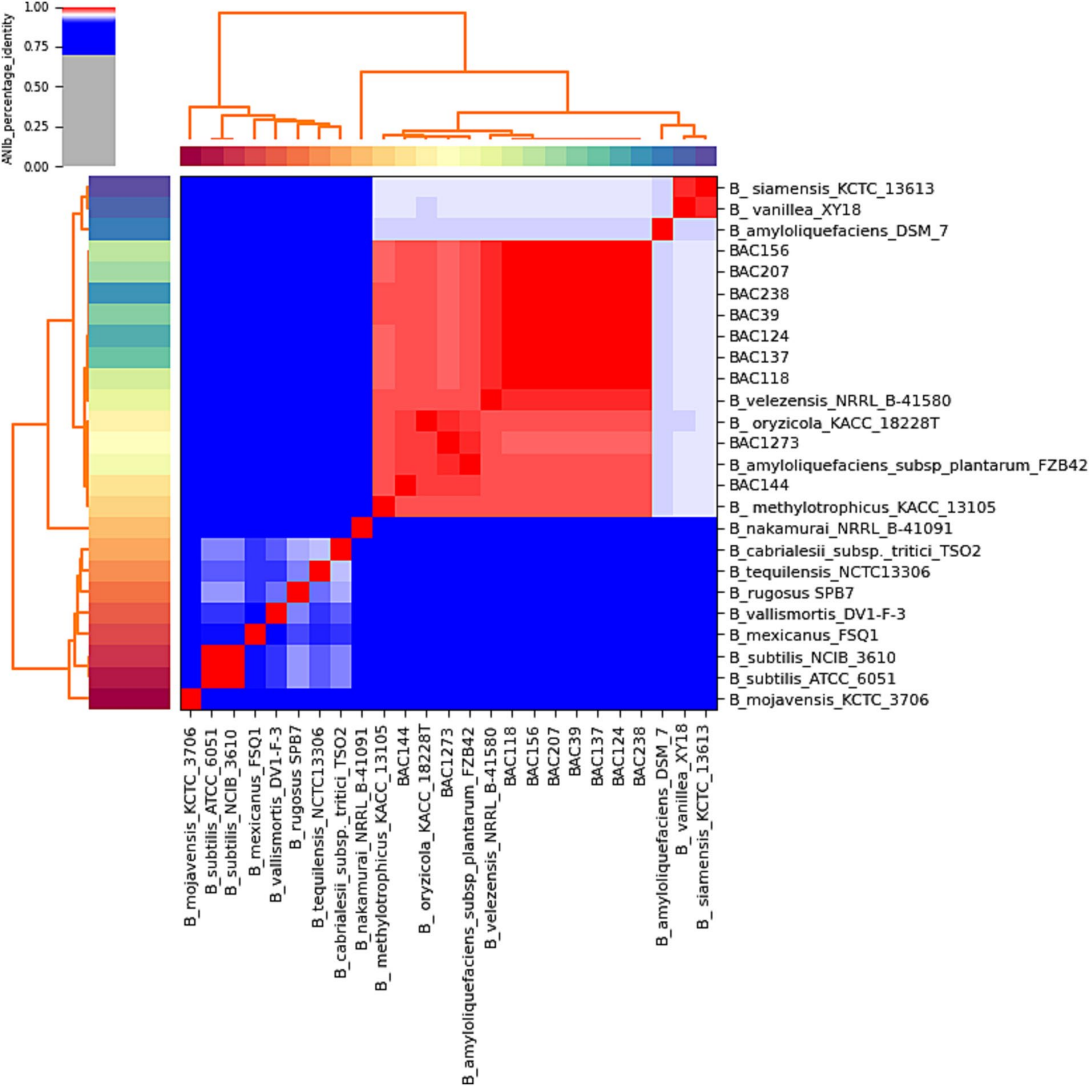
A heatmap of the analysis was carried out to show the similarity percentage, demonstrating very high similarity between the samples isolated in this study and *B. velezensis* strains from the database.

The Partitioned PanGenome Graph of Linked Neighbors (PPanGGoLin) program, a tool for analyzing the pangenome of prokaryotes (Gautreau et al., 2020), was used to analyze the shared gene repertoire of the strains isolated in this study with the species from the NCBI public database comparing with *B. velezensis* (370 complete genomes, accessed 07 August 2024). Additionally, we included strains from the same phylogenetic clade as highlighted in the gray square in Figure 1, ensuring a more refined comparative analysis. In order to check the proportion of genes shared by the bacteria in this study with those available in the database. The program uses a combination of graphical representation and machine learning to categorize genes based on their frequency and distribution, resulting in the following classification: Genes that are persistent and conserved, and which are present in almost all genomes, are associated with essential functions. These are followed by shell genes of intermediate occurrence, which are linked to ecological adaptation or resistance. Finally, there are cloud-specific genes, which are often acquired by horizontal transfer, and which indicate genomic plasticity.

### 2.7 Analyzing synteny and gene similarity

To assess possible gene rearrangements and to analyze collinearity between genes when comparing genomes, the Mauve program v.20150226 (Darling et al., 2004) was used, together with the progressive Mauve algorithm, to analyze gene synteny between the samples isolated and sequenced in this work and the genome identified by GTDB-Tk, *B. velezensis* NRRL B-41580 (GCF_001461825.1), which is also closely related by phylogenetic tree analysis.

In addition, Gegenees v3.1 software was used to obtain a distance matrix related to the species similarity to each other and to understand the differences between the lineages (Ågren et al., 2012). This tool uses BLASTn to perform an alignment of genome fragments of pre-defined sizes of 500 nucleotides and, in the end, produces a heat map showing the similarity between each strain in a range from 0 to 100 percent.

### 2.8 Comparative functional genomics

The program eggNOG-mapper v2 (Cantalapiedra et al., 2021) was performed for functional annotation, orthology assignments, and domain prediction. The PFAM, KEGG, and COG databases were used to describe the exclusive proteins of the groups determined based on the obtained results. To achieve this, the genomic repertoire matrix generated by PPanGGOLiN was used, and the annotation was performed using an in-house script in R. Comparative analysis of functional annotation was based on the results of taxonomic and phylogenomic analyses. A presence/absence-based approach was applied to identify genes exclusively present in each group while being absent in the remaining ones.

## 3 Results

### 3.1 Identification by MALDI-TOF MS

In this study, the utilization of MALDI-TOF MS technology led to the identification of two isolates, designated as genus *Bacillus* (BAC 118 and BAC 207), within the range of 1,717 to 1,819, as specified by the manufacturer. Four strains (BAC 39, BAC124, BAC156 and BAC 238) were unable to be identified, classified as having no peaks found (score < 0), and three (BAC137, BAC144 and BAC1273) exhibited a very low score and were therefore deemed unreliable for identification purposes, the color red is indicative of a low score, with minimal identifying power. Conversely, yellow signifies an intermediate score, yet still not conclusive (Table 2). This method is based on comparing the mass spectrometry profile of the isolate with a reference database. The score reflects the degree of similarity between the spectrum generated by the sample and the profiles present in the database. It was not possible to identify the species of the isolates from these scores.

**Table 2:** Identification of isolates by MALDI-TOF MS.

Isolate	Organism (best match)	Score value
BAC39	No peaks found	<0
BAC118	*Bacillus subtilis*	1.819
BAC124	No peaks found	<0
BAC137	*Bacillus amyloliquefaciens*	1.683
BAC144	*Bacillus mojavensis*	1.569
BAC156	No peaks found	<0
BAC207	*Bacillus mojavensis*	1.717
BAC238	No peaks found	<0
BAC1273	*Bacillus vallismortis*	1.568

The color red indicates a low score, signifying minimal identifying power. Yellow represents an intermediate score, which is still inconclusive.

### 3.2 Quality and contamination

The quality of the assembly, according to the QUAST report, is shown in Table 3. Isolates BAC1273 and BAC207 had the largest and smallest genomes, with 4,176,055 and 4,029,220 base pairs, respectively. All had 100% completeness, between 0.07 and 0.22 contamination by CheckM2, and did not show chimerism as predicted by GUNC (Supplementary Table 3). In addition, the genomes had between ~3,952 and 4,125 coding sequences (CDS), 3 to 5 rRNA genes, and 76 to 84 tRNA genes which were predicted by Prokka.

**Table 3:** Quality metrics for *Bacillus* isolation.

Isolate	Size	Contigs	N50	L50
BAC39	4,029,777	39	527,624	3
BAC118	4,033,356	48	2,031,874	1
BAC124	4,029,940	39	527,724	3
BAC137	4,029,333	42	527,724	3
BAC144	3,992,873	62	450,794	4
BAC156	4,030,244	36	2,031,874	1
BAC207	4,029,220	43	351,435	4
BAC238	4,029,941	39	527,724	3
BAC1273	4,176,055	20	1,064,843	2

### 3.3 Taxonomic identification and typing of genomes

All the strains were classified as *B. velezensis* by GTDBtk, with an ANI value above 95% (Table 4). Two isolates, BAC1273 and BAC144, were classified as *B. amyloliquefaciens subsp. plantarum* with strain *FZB4* (GCF_001461825.1) as reference by TYGS with a dDDH of 91.8%. Nevertheless, the latter strain was reclassified as *B. velezensis* by Dunlap et al. (2016), and by the List of Prokaryotic names with Standing in Nomenclature (LPSN) (Dunlap et al., 2016).^12^ All the other strains were classified as *B. velezensis* with dDDH ranging from 89.3 to 91.8% with high similarity to *strain* NRRL B-41580 (GCF_001461825), which was selected as the reference for the subsequent analyses in this study (Table 4).

**Table 4:** Taxonomic classification of genomes using GTDB-Tk and TYGS.

Name	GTDB-Tk classification	GTDB-Tk fastani	GTDB-Tk fastani_reference	TYGS classification	TYGS dDDH (d4, in %)	TYGS C.I. (d4, in %)
BAC39	*d__Bacteria;p__Bacillota;c__Bacilli;o__Bacillales;f__Bacillaceae;g__Bacillus;s__Bacillus velezensis*	95.0	GCF_001461825.1	*Bacillus velezensis NRRL B-41580*	91.8	[89.7–93.5]
BAC118	*d__Bacteria;p__Bacillota;c__Bacilli;o__Bacillales;f__Bacillaceae;g__Bacillus;s__Bacillus velezensis*	98.89	GCF_001461825.1	*Bacillus velezensis NRRL B-41580*	91.8	[89.7–93.5]
BAC124	*d__Bacteria;p__Bacillota;c__Bacilli;o__Bacillales;f__Bacillaceae;g__Bacillus;s__Bacillus velezensis*	98.91	GCF_001461825	*Bacillus velezensis NRRL B-41580*	91.8	[89.7–93.5]
BAC137	*d__Bacteria;p__Bacillota;c__Bacilli;o__Bacillales;f__Bacillaceae;g__Bacillus;s__Bacillus velezensis*	98.92	GCF_001461825	*Bacillus velezensis NRRL B-41580*	91.8	[89.7–93.5]
BAC144	*d__Bacteria;p__Bacillota;c__Bacilli;o__Bacillales;f__Bacillaceae;g__Bacillus;s__Bacillus velezensis*	98.04	GCF_001461825.1	*Bacillus amyloliquefaciens subsp. plantarum FZB42*	89.3	[86.9–91.3]
BAC156	*d__Bacteria;p__Bacillota;c__Bacilli;o__Bacillales;f__Bacillaceae;g__Bacillus;s__Bacillus velezensis*	98.89	GCF_001461825	*Bacillus velezensis NRRL B-41580*	91.8	[89.7–93.5]
BAC207	*d__Bacteria;p__Bacillota;c__Bacilli;o__Bacillales;f__Bacillaceae;g__Bacillus;s__Bacillus velezensis*	95.0	GCF_001461825.1	*Bacillus velezensis NRRL B-41580*	91.8	[89.7–93.5]
BAC238	*d__Bacteria;p__Bacillota;c__Bacilli;o__Bacillales;f__Bacillaceae;g__Bacillus;s__Bacillus velezensis*	95.0	GCF_001461825.1	*Bacillus velezensis NRRL B-41580*	91.8	[89.7–93.5]
BAC1273	*d__Bacteria;p__Bacillota;c__Bacilli;o__Bacillales;f__Bacillaceae;g__Bacillus;s__Bacillus velezensis*	98.14	GCF_001461825.1	*Bacillus amyloliquefaciens subsp. plantarum FZB42*	91.8	[89.7–93.5]

**Figure 2:**
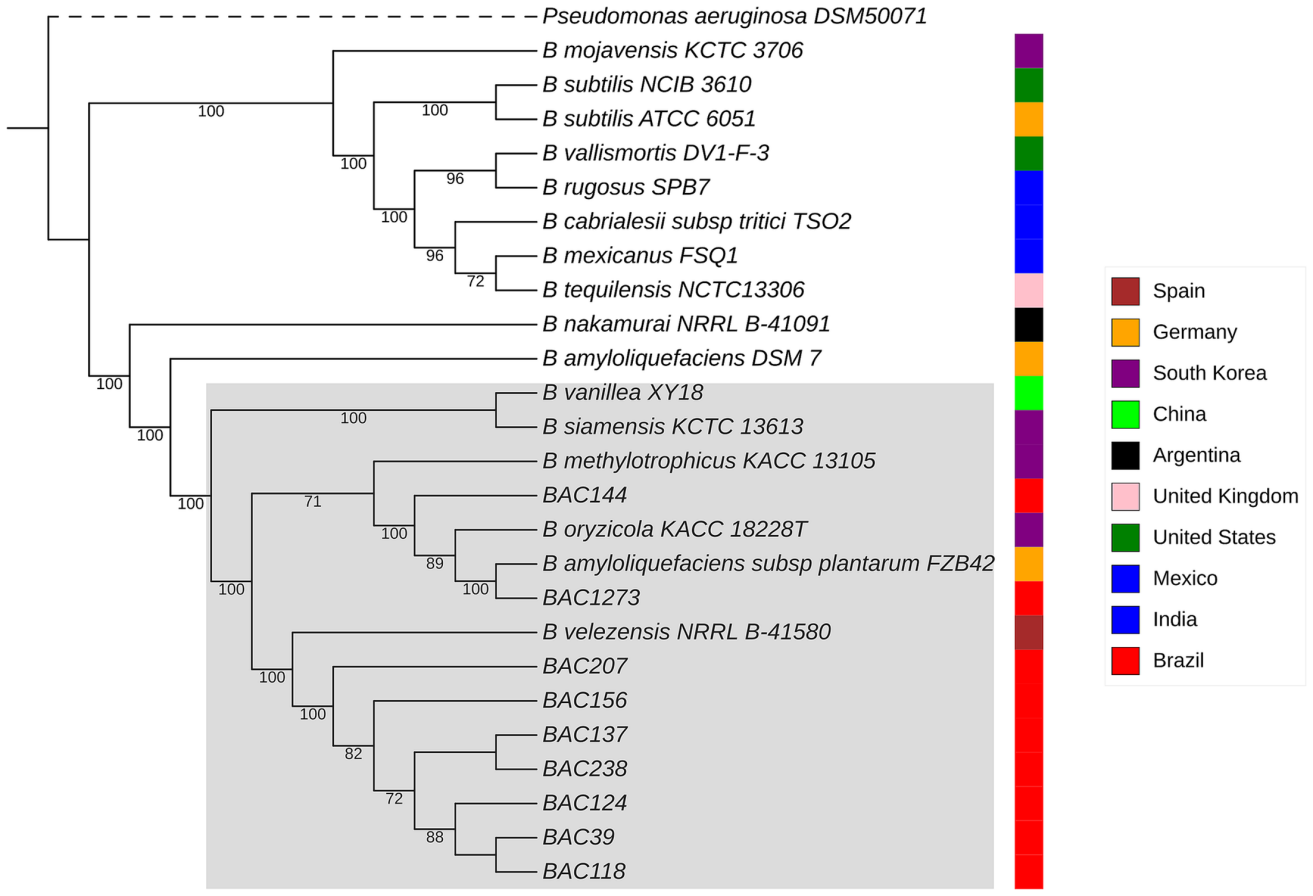
The phylogenetic tree shows the relationships between all related genomes predicted by TYGS for *B. velezensis* strains, along with their geographical locations of isolation. Bootstrap values, based on 1,000 replicates, range from 71 (lowest) to 100 (highest). The same clusters observed in the ANI heatmap can also be visualized here. *B. velezensis* strains that group together are highlighted within a gray square. Additionally, *Bacillus oryzicola* KACC 18228 T, *B. amyloliquefaciens subsp. plantarum FZB42*, and *B. methylotrophicus* were subsequently reclassified as *B. velezensis*.

The genomes identified by TYGS analysis (Supplementary Table 2) were downloaded by RefSeq NCBI in “.fna” format for ANI analyses by pyANI (Figure 2; Supplementary Figure 1). The genomes were compared, including the genome sequence of the 9 strains isolated in this study and 16 identified by TYGS (Supplementary Data 1). The clustering had a high similarity, values greater than 95%, with *B. velezensis* NRRL B-41580, as predicted in the subsequent analyses. In particular, isolates BAC144 and BAC1273 also showed high similarity with *Bacillus oryzicola* KACC 18228 T (GCF_001461835.1), *B. amyloliquefaciens subsp. plantarum* FZB42 and *Bacillus methylotrophicus,* which were subsequently reclassified as *B. velezensis* (Dunlap et al., 2016; Adeniji et al., 2019).^13^ This reclassification is also supported by studies that address issues related to genome identification in databases, particularly those identifications based solely on 16S rRNA sequences (Diabankana et al., 2022).

The isolated strains’ sequence type (ST) was determined using the MLST scheme defined in PubMLST. The strains exhibited a pattern consistent with that of *B. subtilis* for the ST-91 complex, yet they displayed a novel combination of alleles, warranting their classification as a distinct “new_ST” variant. The identified housekeeping genes and their respective alleles were *glpF*, *ilvD*, *pta*, *purH*, *pycA*, *rpoD,* and *tpiA* (Supplementary Table 4). Different alleles were found only in BAC144 and BAC1273.

### 3.4 Phylogenomic analysis and pangenome

The phylogenetic tree was constructed by maximum likelihood with PhyloPhlAn (Asnicar et al., 2020) to determine their genetic relationships using the MUSCLE algorithm (Edgar, 2004) for multiple sequence alignment (MSA) and phylogenetic reconstruction with FastTree.

The first dataset used for the analysis was with strains of the genus *Bacillus* present in NCBI and GTDB, with the two similar predicted by TYGS (25 genomes), as mentioned in Methodology Section 2.1, and with *Pseudomonas aeruginosa* DSM50071^T^ as outgroup (Asnicar et al., 2020). The same pattern was observed in the ANI heatmap, with strains BAC144 and BAC1273 clustering with *B. amyloliquefaciens subsp. plantarum* and the rest with *B. velezensis* NRRLB-41580. In addition, a clade with *B. velezensis*, *B. amyloliquefaciens*, *Bacillus siamensis* and *Bacillus nakamurai* was observed (Supplementary Figure 2). However, the phylogenetic analysis based on the 16S rRNA gene using the TYGS dataset did not provide reliable resolution of the branching positions, as indicated by the low bootstrap values, indicating weak statistical support for the branching positions (Supplementary Figure 3).

The second dataset was based on similar data used in the plane analyses, with the genomes predicted by TYGS, as explained in Section 2.1, and using the same outgroup. The same clustering division between strains was observed, with an additional clade formation with the *B. velezensis* from the database and identified in this study, consisting of *B. velezensis*, *B. amyloliquefaciens*, *B. siamensis*, *B. nakamurai*, *Bacillus vanillea*, *B. methylotrophicus* and *B. oryzicola* (reclassified as *B. velezensis*), which can be seen in Figure 1. The data were analyzed, and it was revealed that the bacterial samples collected from different regions did not group together according to their geographical location. However, the samples isolated in this study grouped together phylogenetically closely, except for BAC144 and BAC 1273, which were close to samples from South Korea (KACC 18228 T) and Mexico (FZB42), respectively.

**Figure 3:**
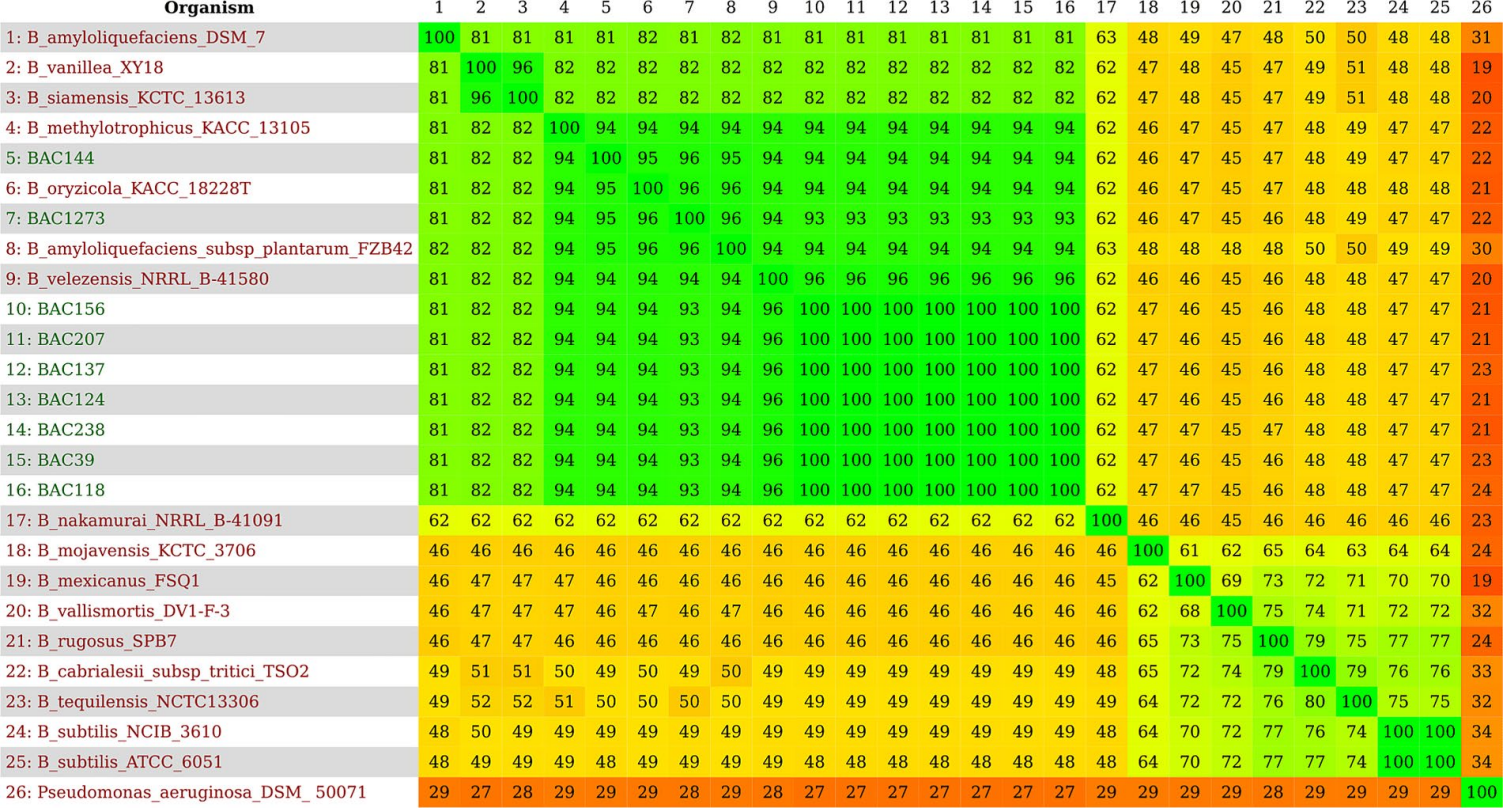
Heatmap with nine genomes identified as *B. velezensis* in this study and 16 representative genomes according to TYGS. In green, a high similarity can be observed, ranging from 81 to 100%; in orange, a median similarity ranging from 46 to 65%; and finally, a low similarity in red, ranging from 19 to 34%.

The 25 genomes used in the subsequent analyses were analyzed to obtain a distance matrix related to the similarity of the species to each other, where greenish colors show high similarity, and reddish colors have low similarity. The genomes that formed a cluster with the isolates from this study in the previous analyses showed the same pattern of similarity with a range of 93 to 100%, and the formation of the same group with high similarity was observed as seen in the phylogenetic trees, with a range of 81 to 96% (Figure 3). The same high similarity between the strains isolated in this study and *B. velezensis NRRL B-41580* was observed in a range of 93 to 94%, presented by BAC144 and BAC1273, up to 96 to 100% with the others (Supplementary Figure 4).

**Figure 4:**
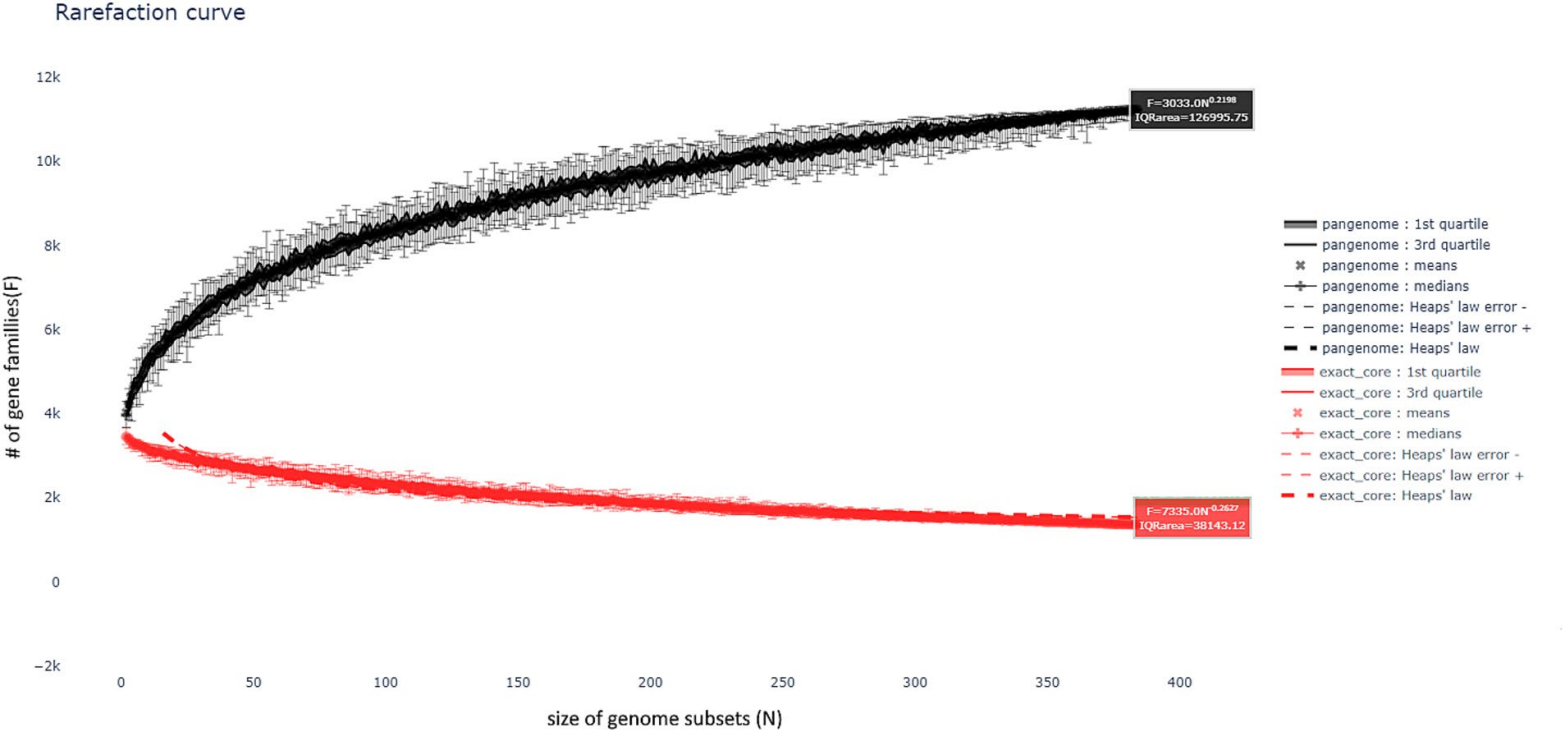
Rarefaction curve of the pangenome of isolated genomes compared to the NCBI database genome of *B. velezensis*, illustrating the open pangenome. The curve shows the increasing number of gene families as more genomes are included, indicating the continuous expansion of gene diversity.

The pangenome analysis revealed the existence of an open pangenome, wherein the number of shared genes among sequenced genomes increases with an *α* value of 0.784. The number of gene families identified in the persistent, shell, and cloud was 3,196, 2,384, and 5,404 genes, respectively. Figure 4 illustrates the rarefaction curve, which demonstrates the open pangenome. It depicts the evolution of the number of gene families as more genomes are incorporated into the pangenome. For each partition, multiple representations of the observed data are provided, including the observed means, medians, 1st and 3rd quartiles of the number of gene families per number of genomes, as well as the best fitting of the data by Heaps’ law, which is commonly used to represent this evolution of diversity in terms of gene families.

### 3.5 Gene synteny

**Figure 5:**
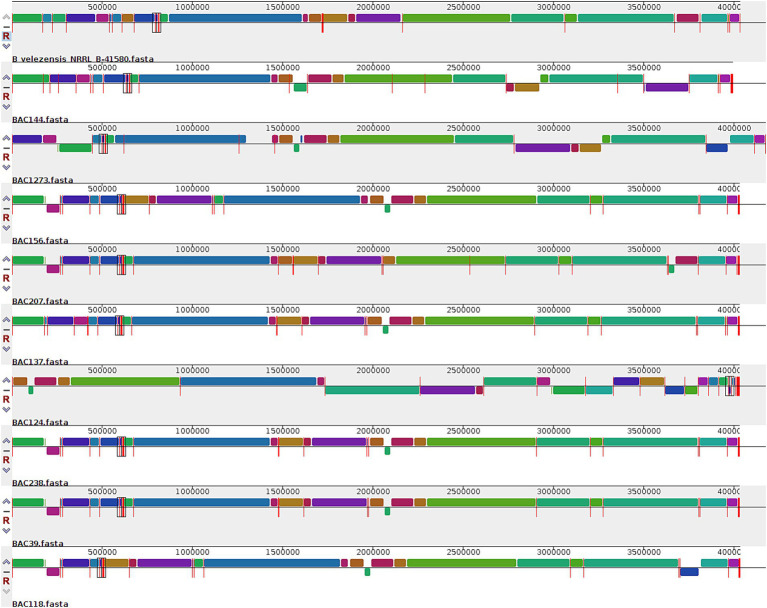
Comparison between the genomes identified in this study and *B. velezensis* NRRL B-41580 according to synteny between the blocks, in which each color corresponds to a specific region that has undergone evolutionary events such as inversions, translocations, or deletions.

A multiple alignment was carried out between the strains isolated in this study and *B. velezensis* NRRL B-41580 from the database, with the high similarity shown in the previous results (Figure 5) and one without this strain (Supplementary Figure 5). We analyzed the synteny between them and whether the one in the database interfered with this collinearity, of which there was no difference. It was possible to observe a high level of conservation between the blocks, with some inversion and translocation processes and some deletion blocks, especially at the end of these genomes. In addition, there was more significant conservation between the strains in this study than in the database. In both analyses, collinearity was also observed between the groups formed: the first formed with only BAC118; another group with BAC124, 137, 238, 1,273, and 39; and one with BAC144, 156, and 207.

### 3.6 Comparative functional genomics

Based on the clustering observed in the phylogenetic tree (Figure 2), a functional analysis of exclusive genes was performed using pangenome data and annotation with eggNOG-mapper. Three groups were analyzed: a subset of seven genomes (BAC156, BAC207, BAC238, BAC39, BAC124, BAC137, and BAC118), BAC144, and BAC1273, each compared to the remaining genomes. Exclusive genes were identified through the gene presence/absence matrix from PPanGGOLiN and annotated using COG, KEGG, and PFAM domains.

**Figure 6:**
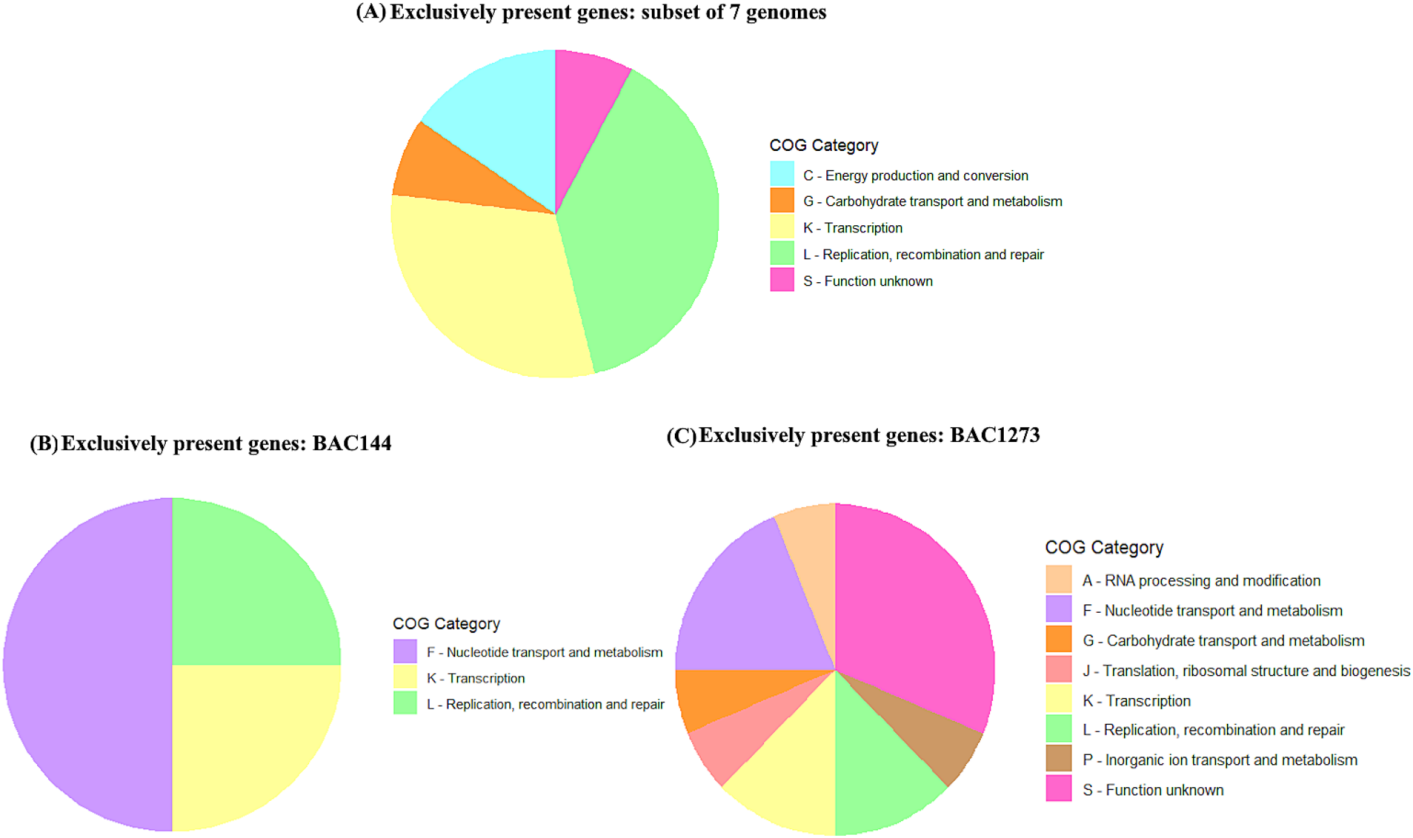
Distribution of COG categories among exclusive genes identified in the subsets analyzed. The three groups include **(A)** the subset of seven genomes (BAC156, BAC207, BAC238, BAC39, BAC124, BAC137, and BAC118), **(B)** BAC144, and **(C)** BAC1273, compared against the remaining genomes. Exclusive genes were classified into their respective COG functional categories.

In the subset of seven genomes, 19 exclusive genes were primarily associated with transcriptional regulation, phage presence, genetic recombination, phosphate metabolism, stress response, and energy metabolism. The most prevalent COG categories were L (Replication, recombination, and repair), K (Transcription), C (Energy production and conversion), S (Function unknown), and G (Carbohydrate transport and metabolism). In the BAC144 genome, four exclusive genes were predominantly linked to transcriptional regulation, nucleotide metabolism, and DNA synthesis, represented mainly by COG categories F, K, and L. In BAC1273, nine genes were identified, which are associated with a variety of functions, including transcriptional regulation, sulfur group transfer reactions, genetic recombination, detoxification, resistance, nucleotide metabolism, DNA synthesis, and carbohydrate metabolism. The most prevalent COG categories were K, L, C, G, and S. A comprehensive summary of these findings can be found in Figure 6 and Supplementary Table 5.

## 4 Discussion

The taxonomic identification of strains by whole genome sequencing is crucial for determining the diversity between bacterial genera and species and differentiating strains. In bacterial research, 16S rRNA sequencing has been extensively studied for its utility in phylogenetic inference. However, this approach has inherent limitations in accurately differentiating the taxonomic group of bacterial lineages. This is because some areas of microorganisms are often conserved, and the forces that shape the evolution of bacterial genomes act with varying degrees of influence on different parts of the genome. Furthermore, it does not provide sufficient evolutionary information (Janda and Abbott, 2007; Gupta et al., 2023). In contrast, the average nucleotide identity (ANI) and dDDH demonstrated superior resolution in differentiating genomes, including those of reference sequences from other species.

In this same context, MALDI-TOF MS technology is widely used for bacterial identification (Han S. S. et al., 2021). However, here, we show that the identification of *B. velezensis* isolates was inconclusive, with very low scores, and a lack of resolution at the strain level. A similar result was obtained with a MLST approach based on genetic diversity of housekeeping genes. The results demonstrated the existence of a novel sequence type (ST), hitherto unrepresented in the PubMLST database. This highlights the necessity for the utilization of whole genome sequencing in conjunction with ANI and dDDH for a robust and more precise identification of bacterial strains. Furthermore, the incorporation of spectral profiles of novel bacterial species and strains into the database could facilitate the accurate identification of bacterial isolates through MALDI-TOF MS technology.

Methods based on whole-genome sequencing data such as ANI and dDDH approaches enabled all nine isolates to be assigned to the *B. velezensis* species (Table 4). Note that two strains (BAC144 and BAC1273) exhibited a high similarity to a *B. amyloliquefaciens subsp. plantarum* strain that was reclassified as *B. velezensis* (Dunlap et al., 2016). The ANI comparison heatmap (Figure 2) demonstrates the clustering of various strains of the *Bacillus* genus, including those isolated and sequenced in this study and belonging to the *B. velezensis* species, as well as strains that have been reclassified within the same species, formerly namely *B. oryzicola, B. amyloliquefaciens subsp. plantarum* and *B. methylotrophicus*. Furthermore, other strains exhibited high similarity to *B. velezensis*, with an ANI of less than 95%, namely *B. siamensis* and *B. methylotrophicus*, which were also reclassified as *B. velezensis* (Dunlap et al., 2016).

The identification and genomic comparison analyses indicate that all strains in this study exhibit high similarity, suggesting that they were derived from a single clone. However, the percentage of similarity was lower for strains BAC144 and BAC1273. This behavior can be explained by analyzing the housekeeping genes, which revealed that only these two strains had different alleles. The sequence type (ST) of the isolated strains was determined using the MLST scheme defined in PubMLST, showing a pattern consistent with the *B. subtilis* ST-91 complex, yet they displayed a novel combination of alleles, warranting their classification as a distinct “new_ST” variant. The identified housekeeping genes and their respective alleles were glpF, ilvD, pta, purH, pycA, rpoD, and tpiA (Supplementary Table 4), with different alleles found only in BAC144 and BAC1273.

In the gene synteny analyses, which demonstrate a high degree of collinearity between the strains in this study (Supplementary Figure 5) and a slightly lower degree of collinearity between the strains and *B. velezensis NRRL B-41580* (Figure 5). The latter was selected for comparison due to its high ANI value compared to the strains in this study. This discrepancy is likely attributable to the fact that the strains were isolated from disparate geographical locations: the strain from the Spanish database and the strains from Brazil.

In the phylogenomic analyses, a maximum likelihood approach was used to investigate the formation of clades between the strains in the study and those in the database. This revealed that the isolated genomes had similar clustering behavior with the same *Bacillus* strains. A monophyletic clade was formed between seven of the strains and another between two of them. These were the same strains that clustered with *B. amyloliquefaciens subsp. plantarum* in all the analyses (Figure 1; Supplementary Figure 2). These were BAC 144 and 1,273. A similar pattern is evident in the similarity heatmap generated using the distance matrix (Figure 3; Supplementary Figure 4). Interestingly, the data revealed that the bacterial samples collected from different geographical regions did not group together based solely on their location. However, the samples isolated in this study clustered phylogenetically closely, except for BAC144 and BAC1273. BAC144 clustered closely with *B. velezensis* strains from South Korea (KACC 18228 T), while BAC1273 showed close phylogenetic ties with a strain from Mexico (FZB42).

The phylogenetic tree constructed using 16S rRNA gene sequences did not provide a reliable resolution, suggesting that the 16S rRNA gene alone may not be sufficient for precise taxonomic placement (Supplementary Figure 3). The lack of strong statistical support in the tree further reinforces the need for whole-genome-based approaches to achieve a more robust classification. Moreover, previous studies have discussed the limitations of using 16S rRNA for analyzing evolutionary relationships within the *Bacillus* genus, as this approach may not always yield consistent or conclusive results (Alcaraz et al., 2010; Velsko et al., 2019; Hassler et al., 2022).

The pangenome of the *B. velezensis* strains isolated in this study was analyzed and compared with the reference genomes in the NCBI database. The results revealed the presence of an open pangenome (Figure 4). This phenomenon is distinguished by the sustained growth in the number of gene families as the analysis incorporates an increasing number of genomes. An open pangenome indicates that, as the number of sequenced genomes increases, the genetic diversity observed continues to grow, as evidenced by the corresponding increase in the number of shared genes. With regard to the strains isolated in this study, it is evident that the number of genes shared between the isolates and the genomes in the NCBI database continues to increase. This suggests a considerable degree of genetic diversity within the *B. velezensis* populations, including previously reclassified variants such as *B. amyloliquefaciens subsp. plantarum* and *B. methylotrophicus*. These findings are consistent with the hypothesis that the *B. velezensis* pangenome is not yet fully closed, but is still dynamic, allowing for the acquisition of new genes as more strains are sequenced.

A comparative analysis of the number of genes shared between the isolated strains and the reference genomes in the database indicates that, while substantial similarities exist, some of the isolated strains exhibited slightly greater genetic diversity. This is exemplified by strains BAC144 and BAC1273. These strains may represent significant genetic variations within the *B. velezensis* species. This pattern was also observed in the phylogenomic analyses, which demonstrated a distinct grouping of these strains, indicating that they may derive from a distinct clone.

As illustrated in Figure 4, the pangenome rarefaction curve demonstrates that the diversity of gene families continues to expand with the addition of new genomes, thereby supporting the concept of an open pangenome. This phenomenon also underscores the necessity of using whole genome sequencing in conjunction with alternative identification techniques, such as ANI and dDDH, for a more precise characterization of strains and to enhance comprehension of the phylogenetic relationships between disparate isolates. This type of analysis is fundamental to understanding the differences and similarities between isolates and reference strains, which has direct implications for the taxonomic classification and biotechnological potential of the strains under study.

Building upon these taxonomic and phylogenomic insights, a deeper functional analysis was performed to investigate the diversity of exclusive gene repertoires within the three identified groups: Subset 7 (BAC156, BAC207, BAC238, BAC39, BAC124, BAC137, and BAC118), BAC144, and BAC1273 (Figure 6; Supplementary Table 5). These analyses revealed distinct adaptations and ecological roles reflected in the predominant COG categories and exclusive gene functions. For Subset 7, the exclusive genes highlight adaptations related to genetic recombination and mobility, such as the integration of mobile genetic elements (e.g., phages and plasmids) through genes like *yqaS* (DNA packaging) with PFAM domain of phage terminase (Médigue et al., 1995; Kimura et al., 2010) and *RecU* (resolution of Holliday junctions) (Cañas et al., 2008). Also, *yobL* (nuclease activity), that is a toxin-immunity that module enhances competitive fitness during biofilm formation by mediating strain segregation and preventing conflict, it ensures survival under competitive conditions (Holberger et al., 2012). The COG category L (Replication, recombination, and repair) predominates, underscoring the genetic flexibility of this group. Additionally, genes like *phoD* (phosphate metabolism) (Mingchao et al., 2010; Huang et al., 2024) and *ydhN3* (carbohydrate transport) (Mingchao et al., 2010) reflect metabolic adaptations, with significant representation in COG categories C (Energy production and conversion) and G (Carbohydrate transport and metabolism). The presence of stress response genes, such as *csbD*, and defense mechanisms, including *yhdJ* (DNA methylation) (Kunst et al., 1997; Adhikari and Curtis, 2016), suggests that this group is well-adapted to environmental challenges, with functions aligned to COG categories S (Function unknown) and K (Transcription).

In contrast, BAC144 exhibited exclusive genes focused on nucleotide metabolism and DNA synthesis, as exemplified by *yncF* (dUTPase domain) (Sznyter et al., 1987; Dervyn et al., 2023) and *nrdE* (ribonucleotide reductase), which maintain genomic stability. The COG category F (Nucleotide transport and metabolism) was particularly enriched, reflecting the group’s emphasis on metabolic precision and DNA replication. Additionally, the gene licT (transcriptional antiterminator) highlights the importance of dynamic transcriptional regulation, supported by genes in COG category K (Transcription). The presence of *ddeI* (DNA methyltransferase) further suggests active epigenetic defense mechanisms, aligning with COG category L (Sznyter et al., 1987). For BAC1273, the exclusive gene repertoire indicates a strong focus on genome defense and environmental adaptation. Genes such as *yhdJ* (methyltransferase) and *mcrA* (restriction endonuclease) point to robust systems for protection against phages and other mobile genetic elements (Mulligan and Dunn, 2008; Bourgeois et al., 2022). This is complemented by the presence of phage-related genes (Phage_capsid and Transposase IS66), indicating interactions with mobile genetic elements. The COG categories K (Transcription) and L (Replication, recombination, and repair) predominate, reflecting the group’s focus on genomic stability and transcriptional control. Additionally, metabolic flexibility is suggested by genes like *dut* (dUTPase) which plays an essential role in nucleotide metabolism, and *yrkH* (Rhodanese) associated with catalytic functions related to sulfur group transfer, classified under COG categories F and P, respectively and indicate the ability to maintain a stable and efficient genome, even under challenging environmental conditions, and provide an advantage in agricultural soils or other environments rich in sulfur and related compounds, enhancing bacterial survival and competitiveness (Huang et al., 1999; Adhikari and Curtis, 2016; Tang et al., 2023; Sisodia et al., 2024). As well as the presence of *exuT* (carbohydrate transport) under COG category G. The gene *xpaC*, associated with detoxification of halogenated compounds (Huang et al., 1999; Hassan, 2021), highlights a unique adaptation to toxic environments, reinforcing the group’s ecological versatility.

These findings align with the phylogenomic analyses, which revealed distinct clustering patterns for the isolates. While most strains formed a cohesive monophyletic clade, BAC144 and BAC1273 consistently grouped separately in all analyses. This divergence is consistent with the functional differences observed in their exclusive gene repertoires, indicating distinct ecological roles and adaptive strategies. The results underscore the importance of integrating functional and phylogenomic analyses to fully understand the genetic diversity and adaptive potential of bacterial populations. Taxonomic studies are of great importance for the identification of strains based on their genomes, given that the taxonomy of prokaryotes has undergone significant changes and reclassifications as a result of the advancement of more sophisticated technologies (Ferraz Helene et al., 2022). Several reclassifications have been proposed for the genus *Bacillus* following comparative phylogenomic analysis, including the reclassification of *B. cereus* and *B. subtilis* (Xu and Kovács, 2024). This study presents the classification of several *B. velezensis* strains and their high similarity to strains reclassified to the same species.

A further study by Fan et al. (2017) and Elshaghabee et al. (2017) demonstrates the formation of an operational group of *B. amyloliquefaciens*, as were the taxonomic status of related strains of *B. amyloliquefaciens*, as evidenced by comparative and phylogenomic analyses. The operational group *B. amyloliquefaciens* comprises the following strains: *B. amyloliquefaciens subsp. plantarum FZB42*, *B. velezensis*, *B. methylotrophicus KACC 13105*, *B. siamensis KCTC 13613,* and *B. amyloliquefaciens DSM7,* the same strains used in this study. It should be noted that this classification does not designate these strains as a species but rather as a group. The same strains used in this study were also used to demonstrate the formation of the same monophyletic group, with a high degree of similarity between them. This suggests a reclassification of these strains as the same species due to the high degree of similarity in this study and similar applications in the agricultural industry. For example, *B. velezensis* is a heterotypic synonym of *B. methylotrophicus*, *B. amyloliquefaciens subsp. plantarum*, and *B. oryzicola*, which are used for the control of plant fungal diseases (Ngalimat et al., 2021).

The identification of these strains is crucial for a more comprehensive understanding of their potential applications and contributions in fields such as microbiology, genetics, bioinformatics, and biotechnology. The results of the comparative analysis revealed a close grouping and similarity between the isolates and the strains of the previously mentioned operational group, as well as between the isolates and *B. vanillea* XY18 and *B. oryzicola* KACC 18228 T. A comparative study by Xu et al. (2020) demonstrates and compares the applicability of strain FZB42 as a biofertiliser, biocontrol and probiotic (Xu et al., 2020). The study also considers the production of secondary metabolites and the antifungal and antibacterial activities of the strain. Additionally, other studies have demonstrated its capacity for fengycin production (Medeot et al., 2023) and probiotic activity (Reva et al., 2019). Moreover, our studies revealed that the FZB42 strain exhibited a high degree of similarity to the isolates.

*Bacillus velezensis* is a bacterium of significant importance in several industrial sectors, including food, farming, and biomedical. It has a variety of applications, including as a potential probiotic. Classifying these strains is essential to subsequently evaluate their gene repertoire for possible applications (Khalid et al., 2021; Huang et al., 2023; Sam-on et al., 2023).

## 5 Conclusion

*Bacillus velezensis* is an important specie but its identification is not yet correctly identified by MALDI-TOF MS. Identifying and classifying bacterial strains of this species is essential to facilitate differentiation from other strains within the *Bacillus* genus. This is due to the advent of more robust technologies for taxonomic analysis. The present study identified nine strains belonging to *B. velezensis*. It demonstrated the necessity for reclassifying other species of the same genus through next-generation sequencing and comparative genomic and phylogenomic analyses. Additionally, the functional annotation of exclusive genes provided critical insights into the ecological roles and adaptive capacities of the strains. The observed functional diversity, reflected in the predominant COG categories, revealed specific adaptations across the analyzed groups. Future analyses will be necessary to characterize each of these strains to better understand the applications of these isolates, given the high applicability of the species.

## Data Availability

The datasets presented in this study can be found in online repositories. The names of the repository/repositories and accession number(s) can be found in the article/Supplementary material.

## Footnotes

^1^ https://tygs.dsmz.de

^2^ https://github.com/OpenGene/fastp

^3^ https://quast.sourceforge.net/

^4^ https://github.com/chklovski/CheckM2

^5^ https://github.com/grp-bork/gunc

^6^ https://github.com/tseemann/prokka

^7^ https://tygs.dsmz.de

^8^ https://github.com/Ecogenomics/GTDBTk

^9^ https://github.com/EnzoAndree/FastMLST

^10^ https://github.com/biobakery/phylophlan

^11^ https://itol.embl.de

^12^ https://lpsn.dsmz.de/subspecies/bacillus-amyloliquefaciens-plantarum

^13^ https://lpsn.dsmz.de/species/bacillus-oryzicola
