# Supplementary material for: The research on the identification, taxonomy, and comparative genomics analysis of nine Bacillus velezensis strains significantly contributes to microbiology, genetics, bioinformatics, and biotechnology
Source: Front Microbiol. 2025 Mar 19;16:1544934. doi: 10.3389/fmicb.2025.1544934 (PMC11962042; doi:10.3389/fmicb.2025.1544934)
Supplement: Supplementary file 1 [file Data_Sheet_1.docx]

Supplementary Material

# Supplementary Data

**Supplementary Data 1:** Taxonomic classification of genomes using the Type Strain Genome Server.


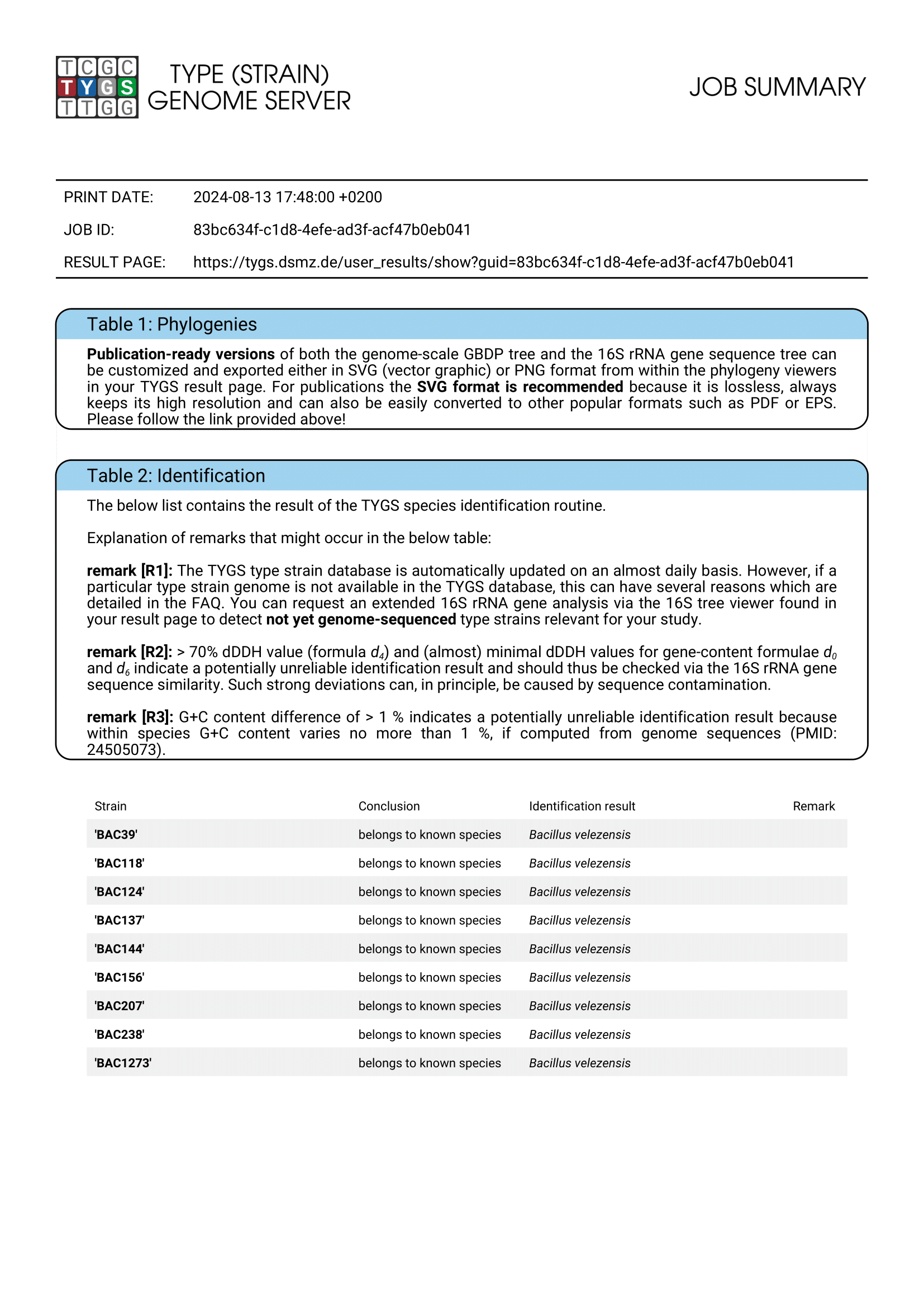


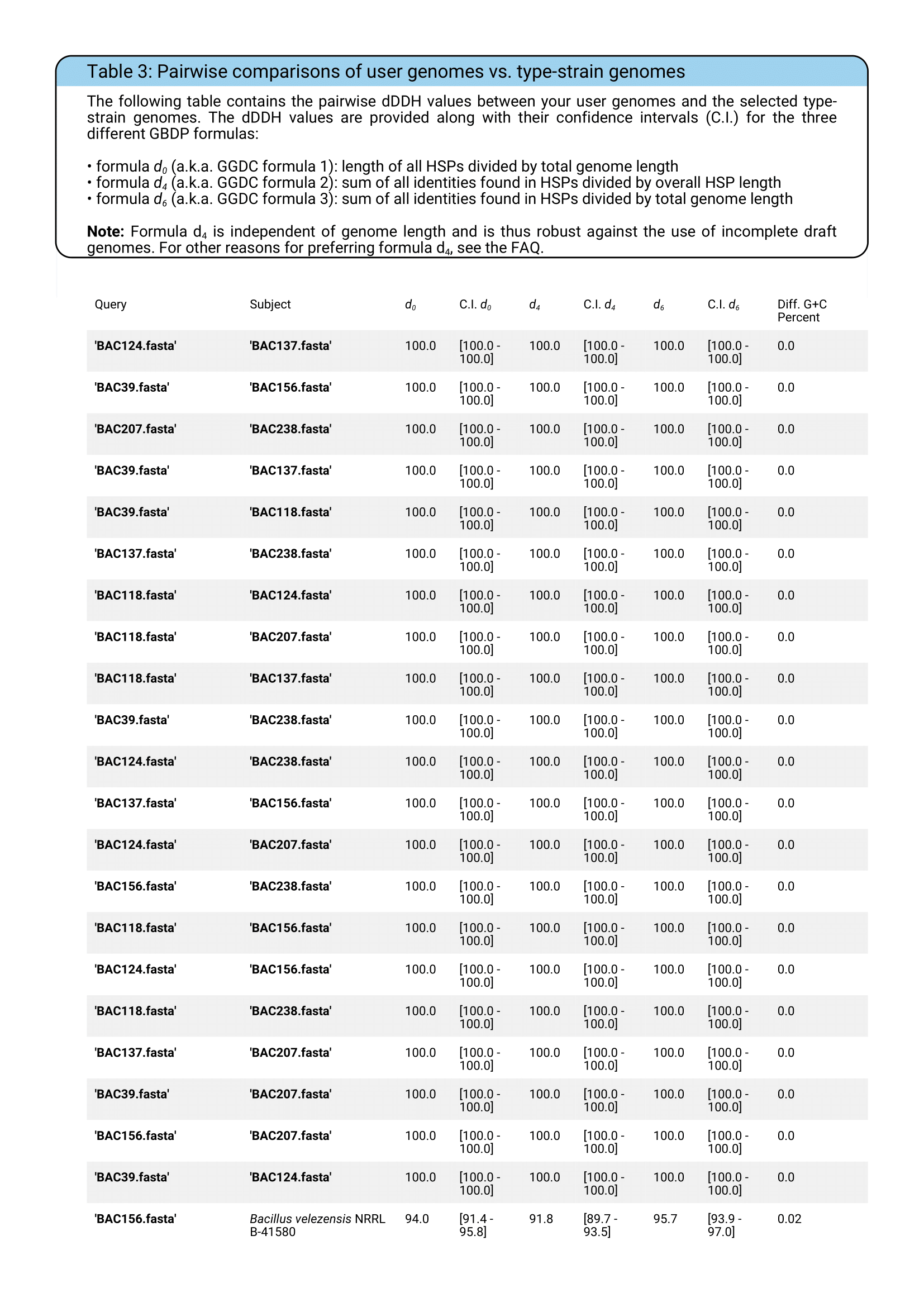

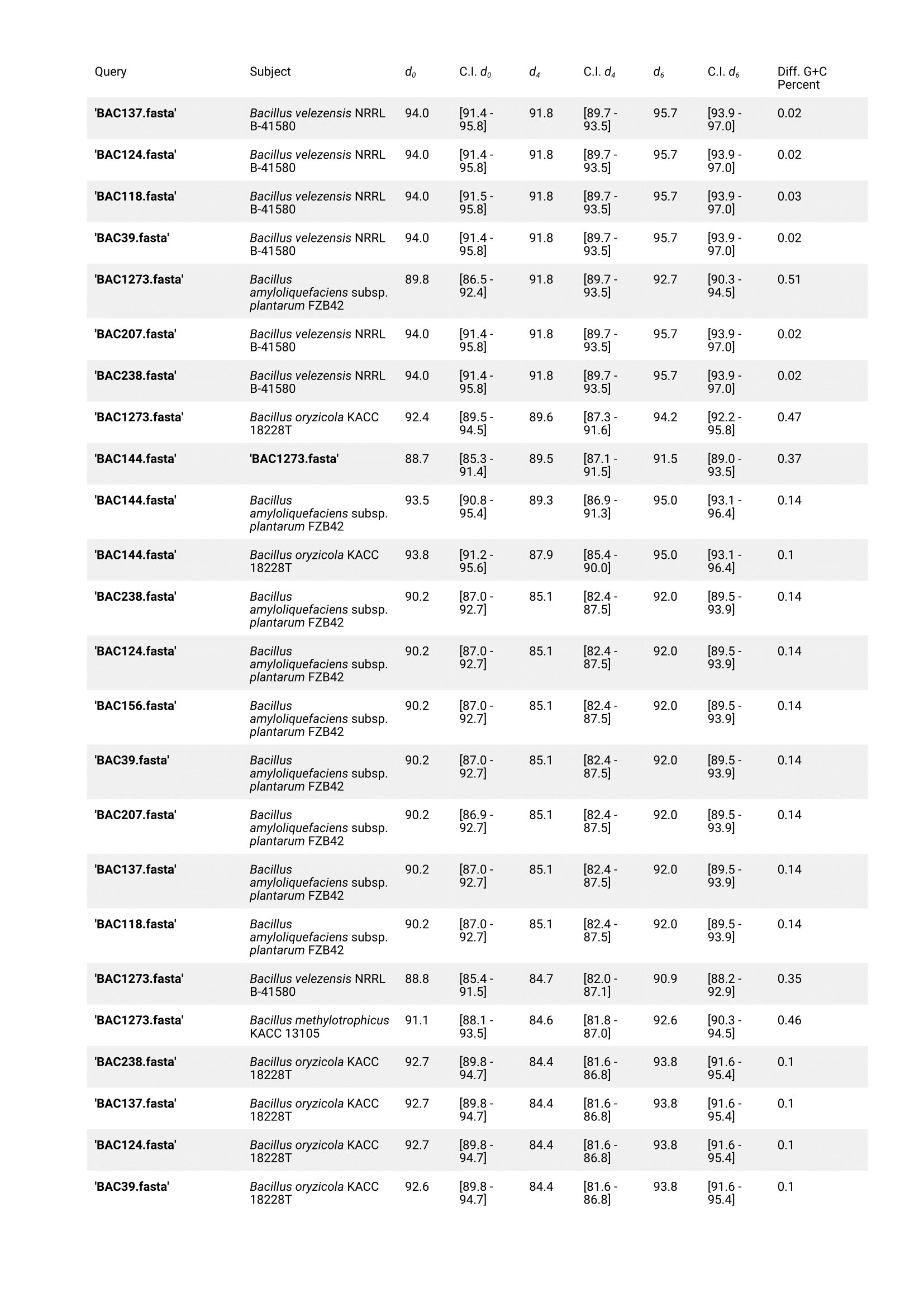

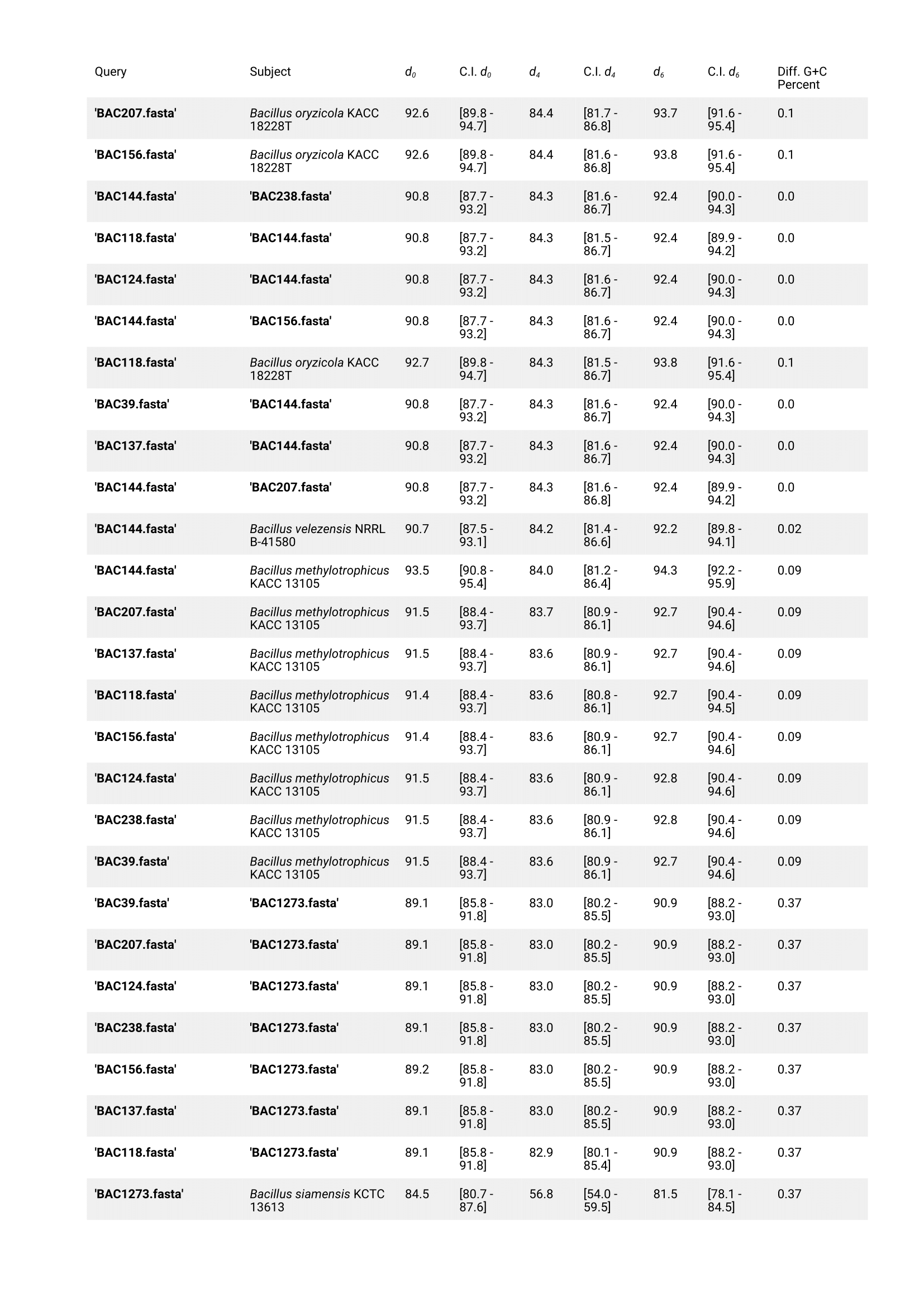

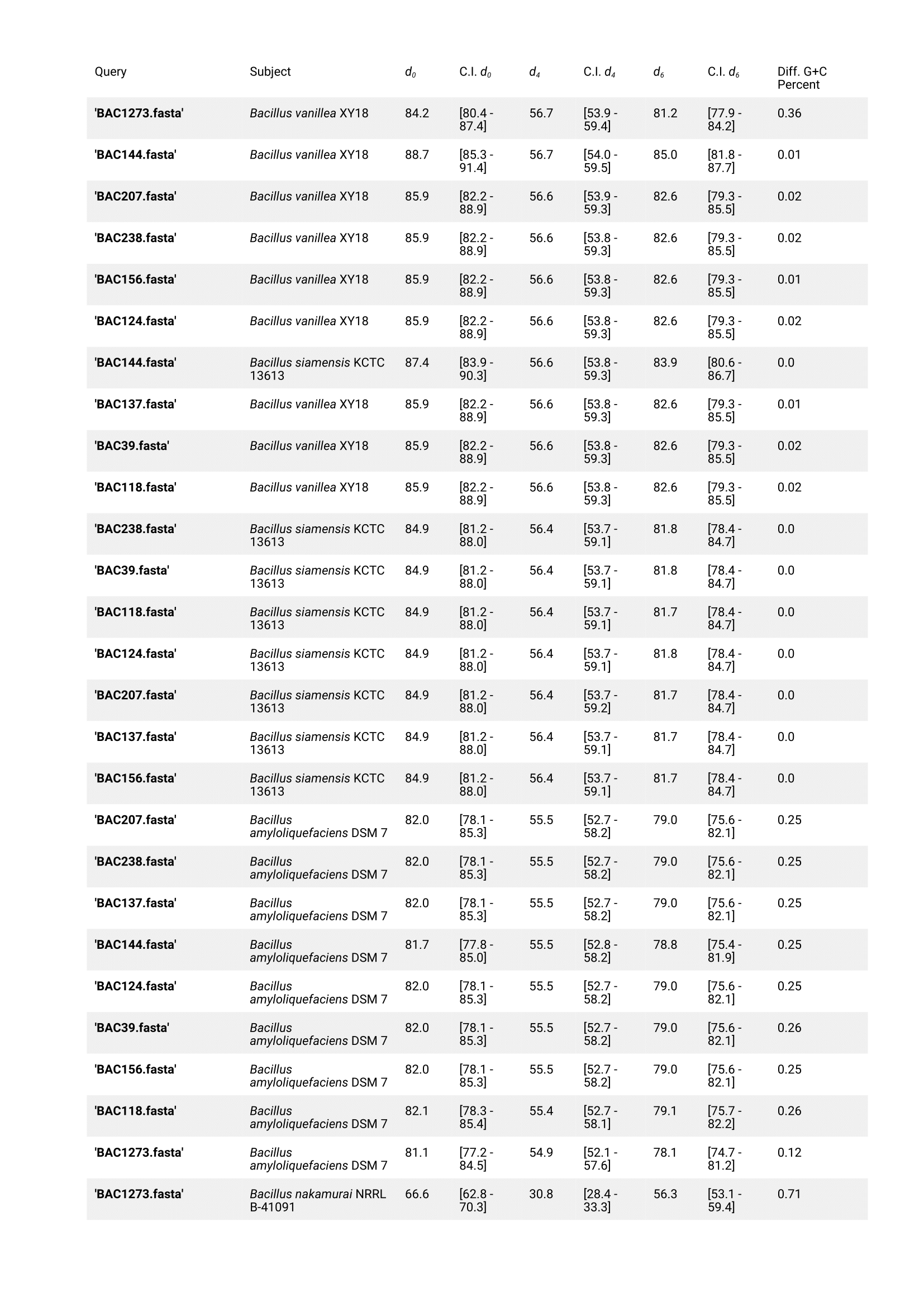

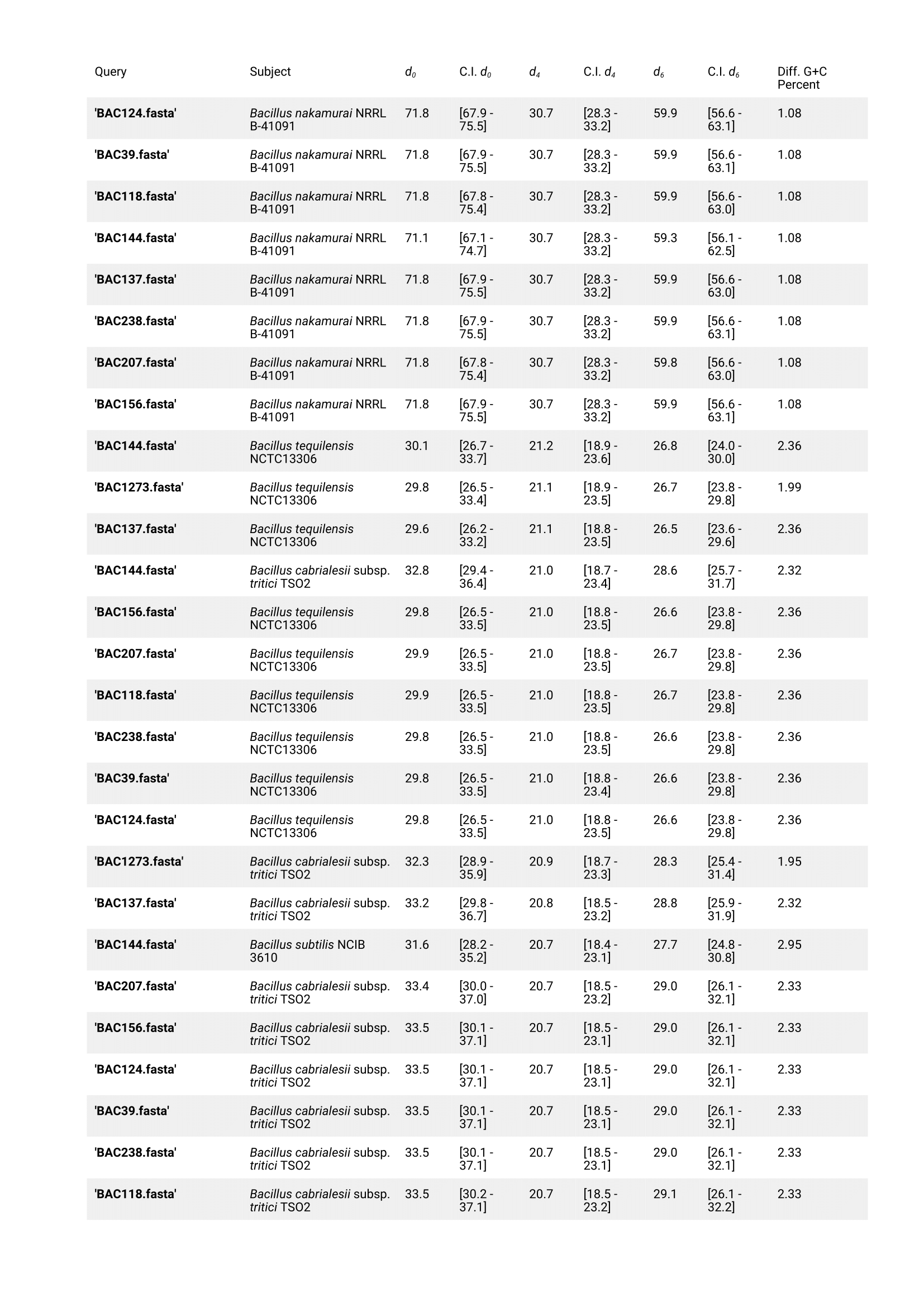

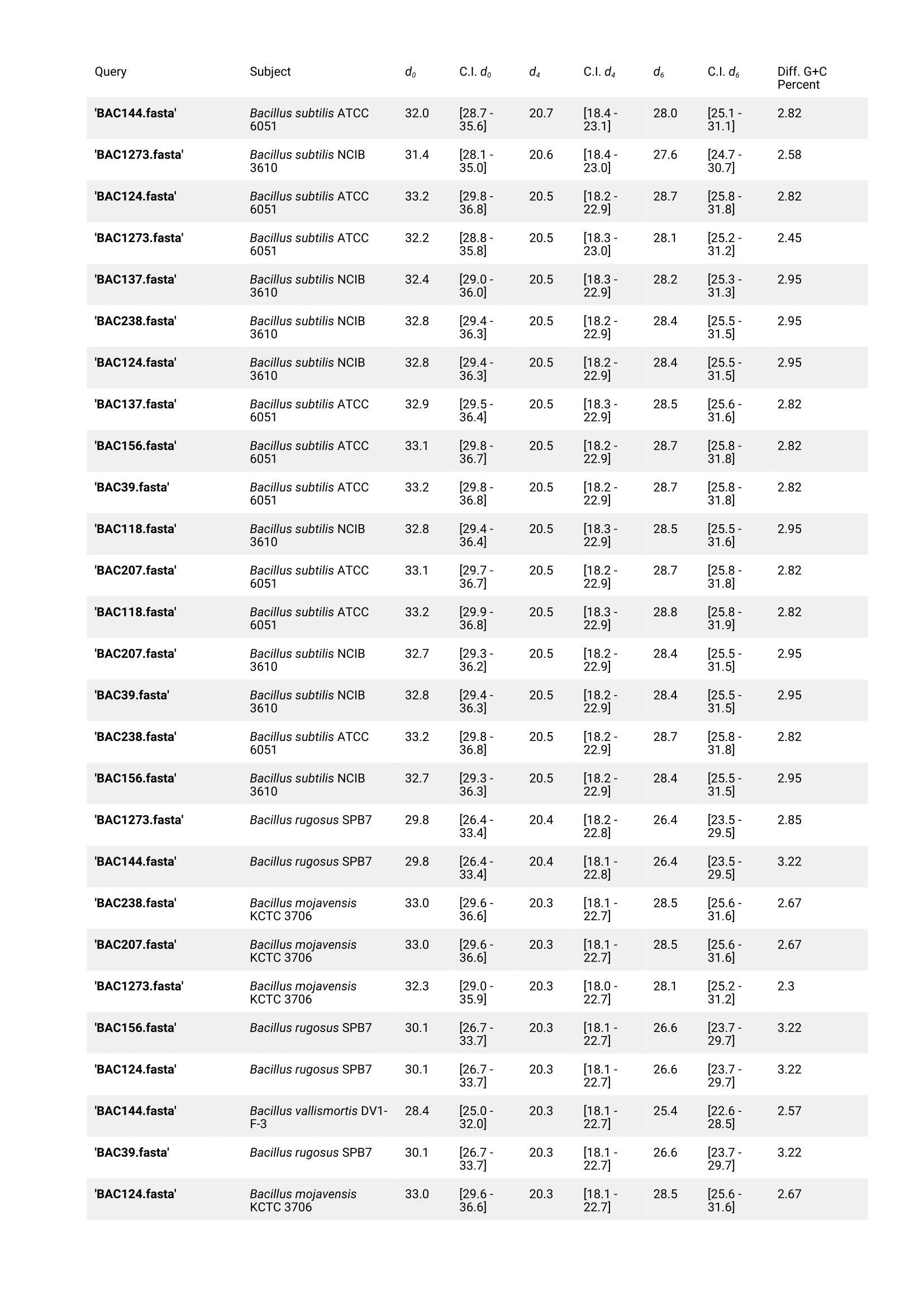

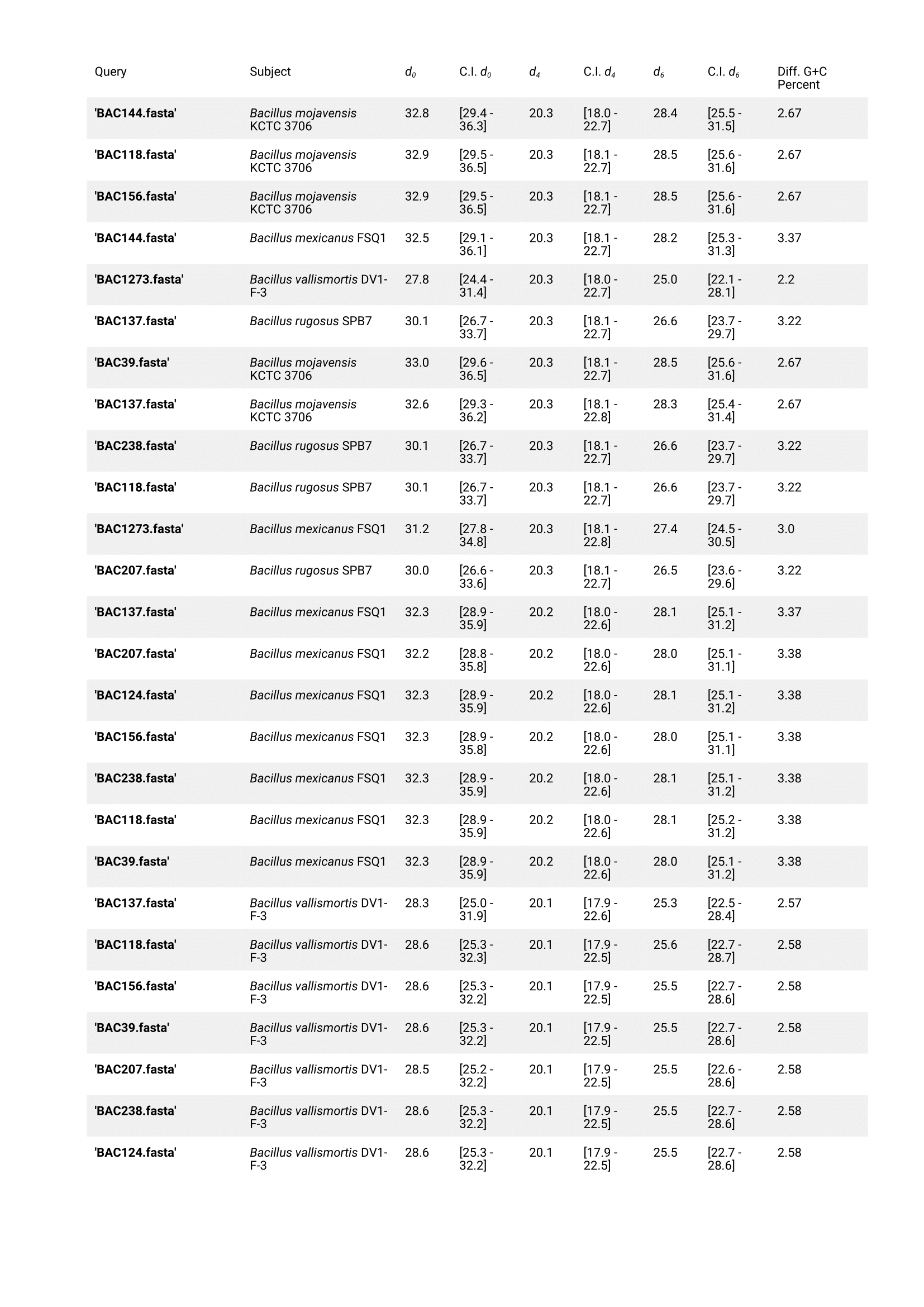

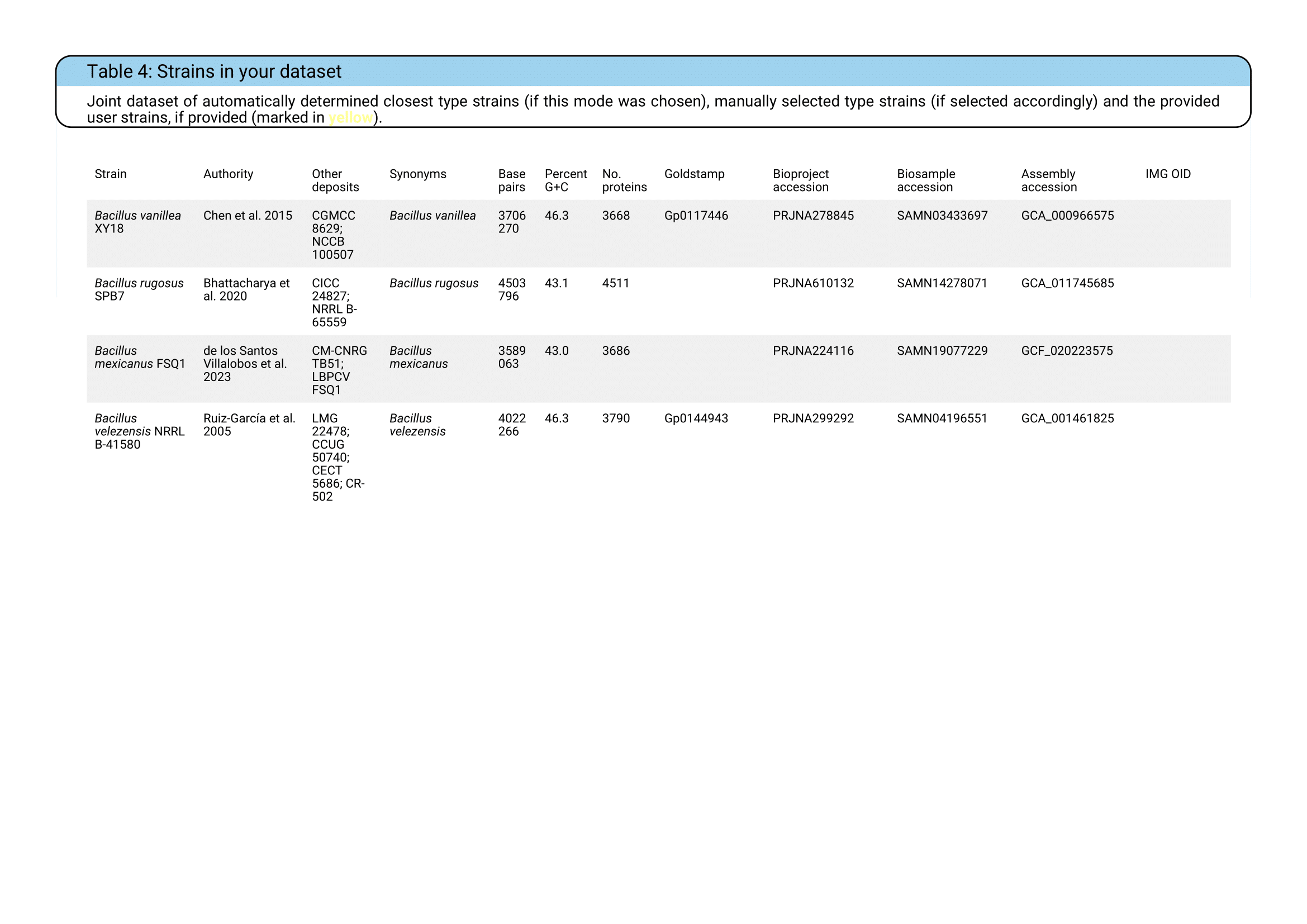

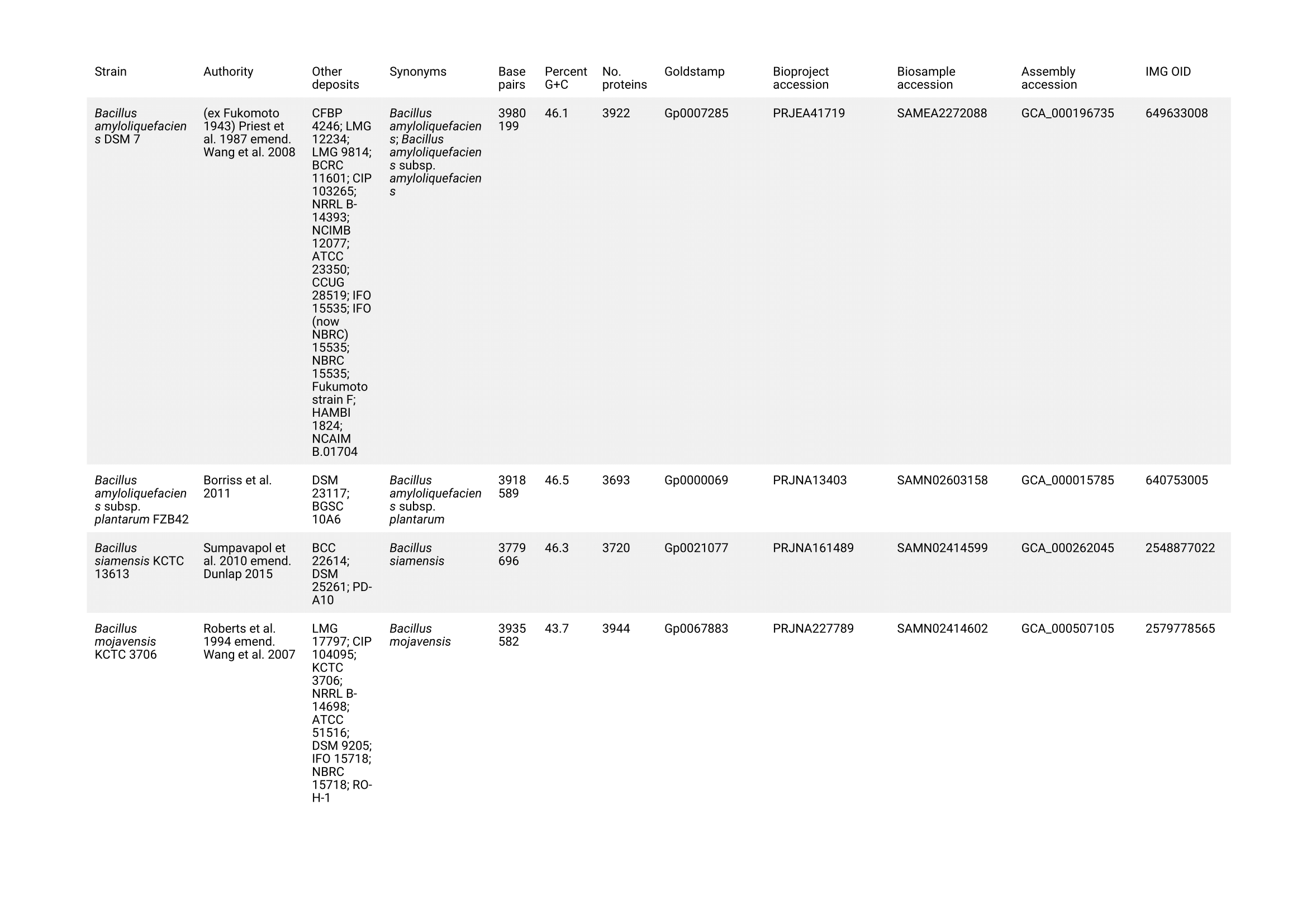

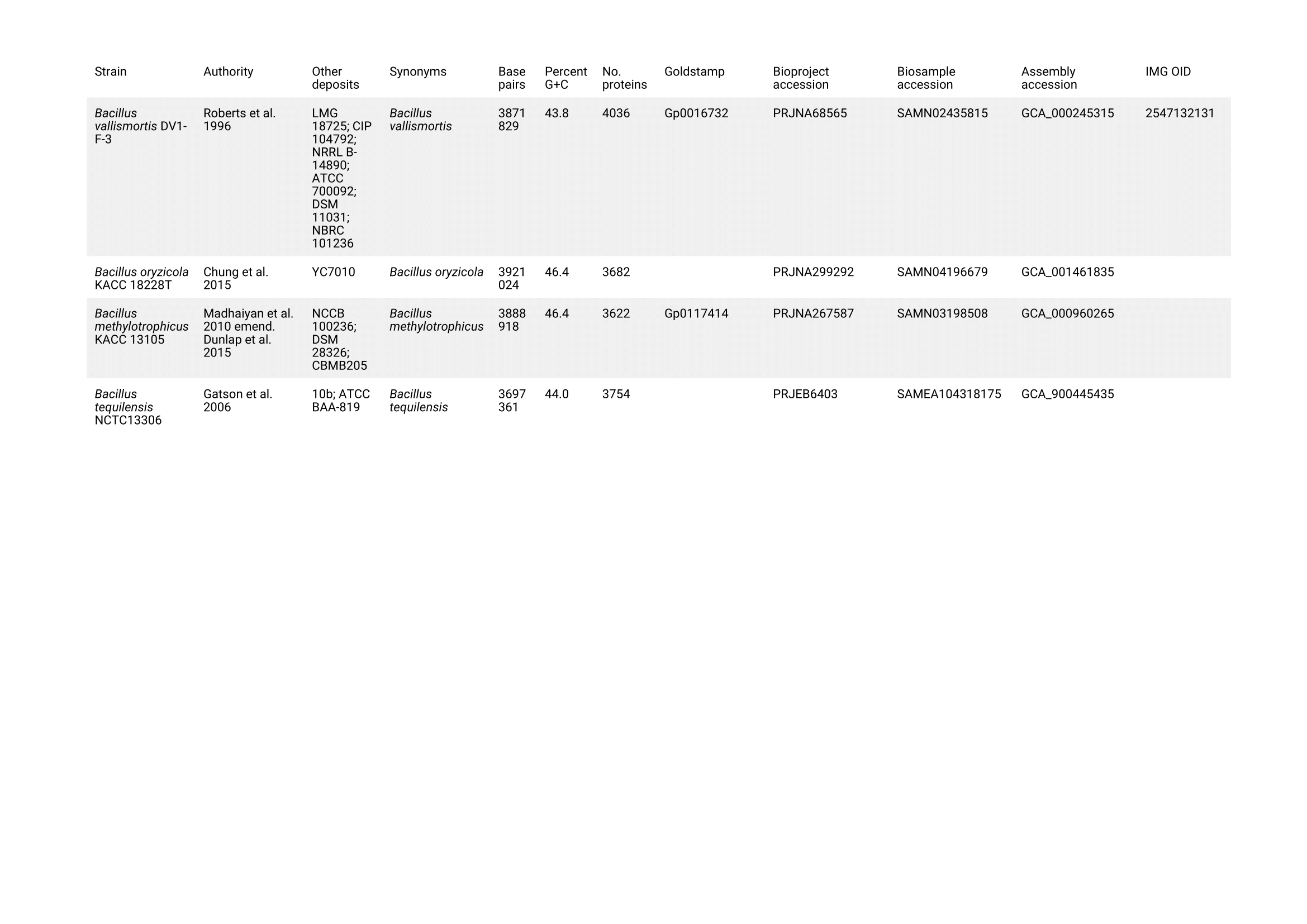

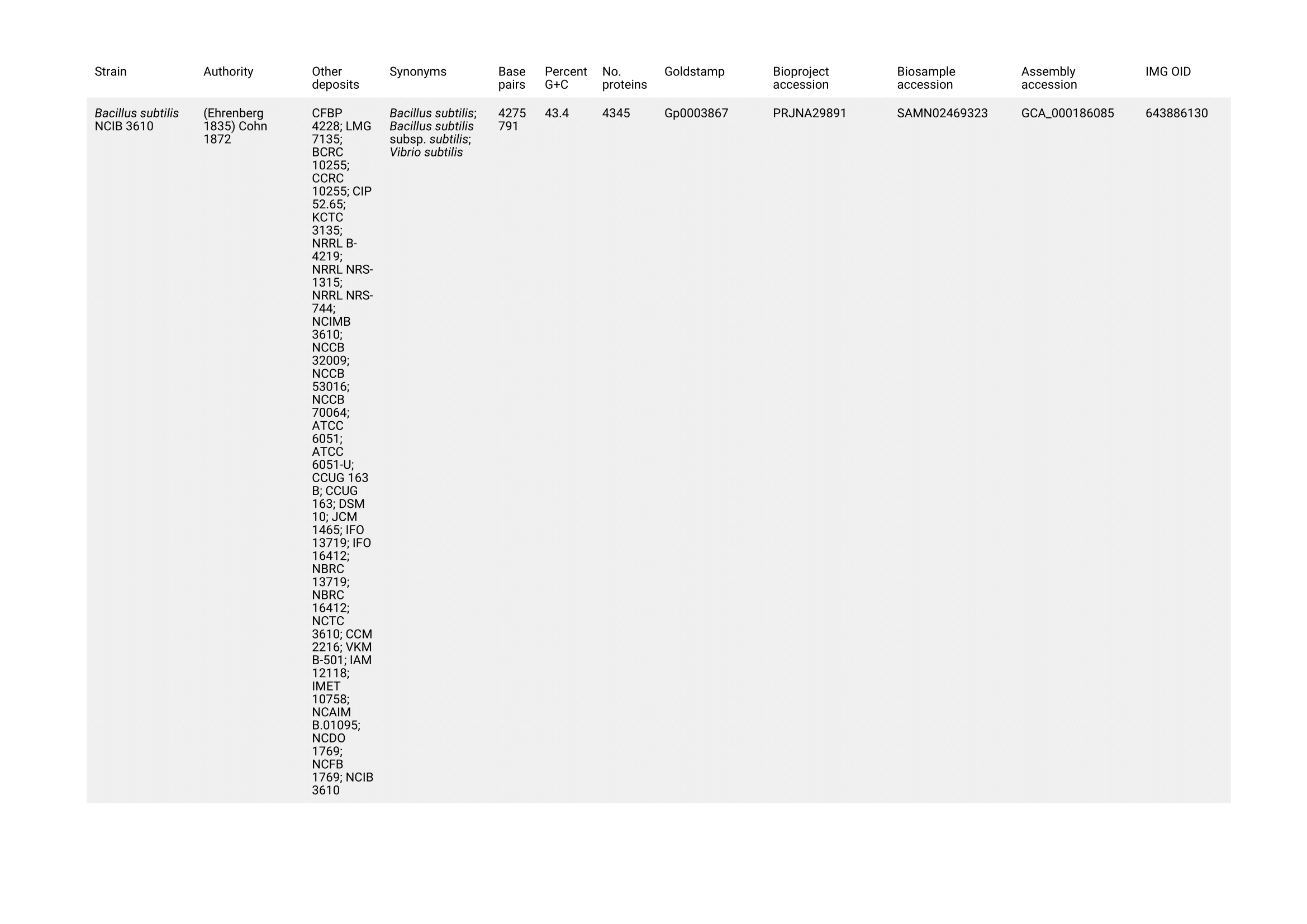

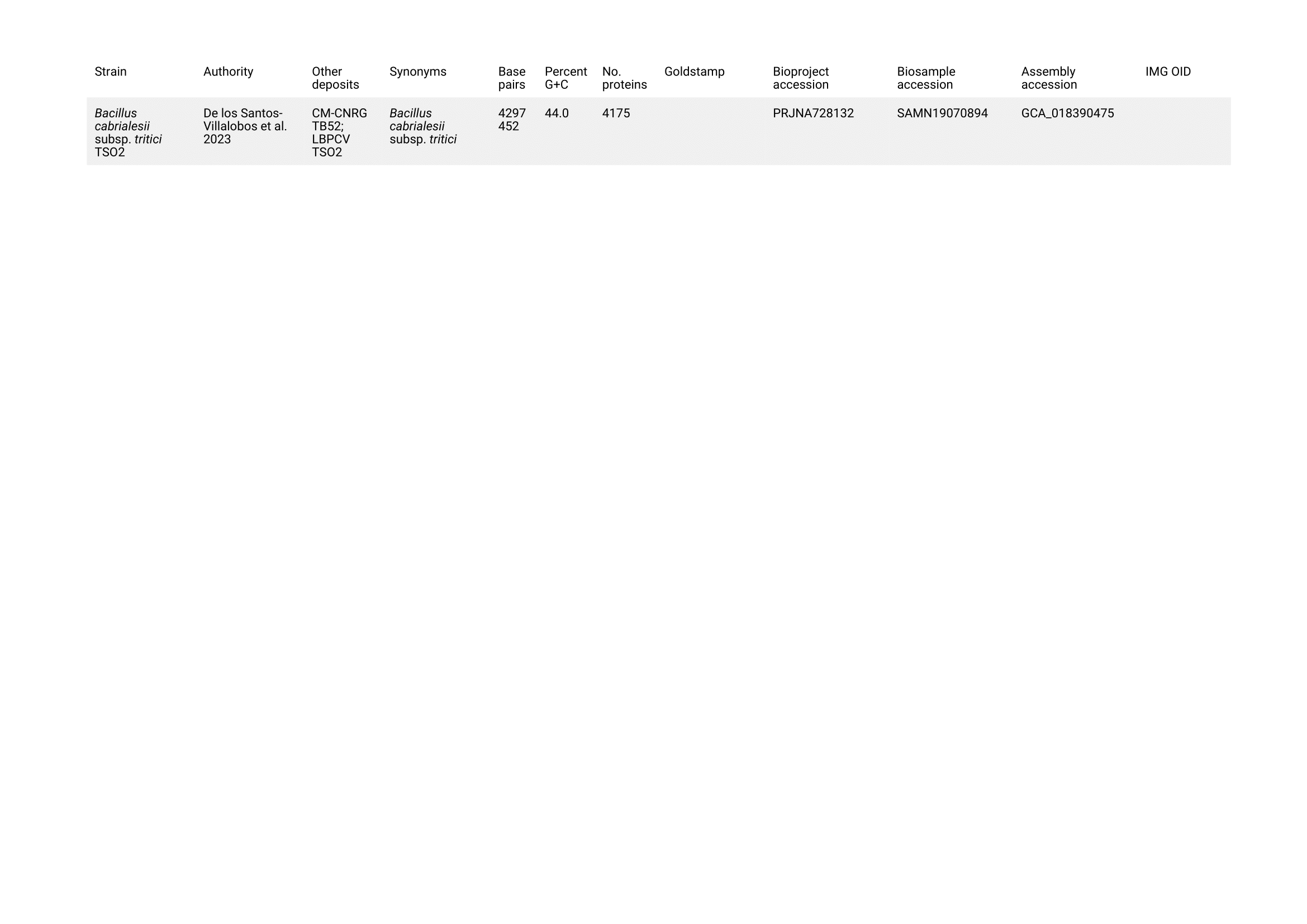

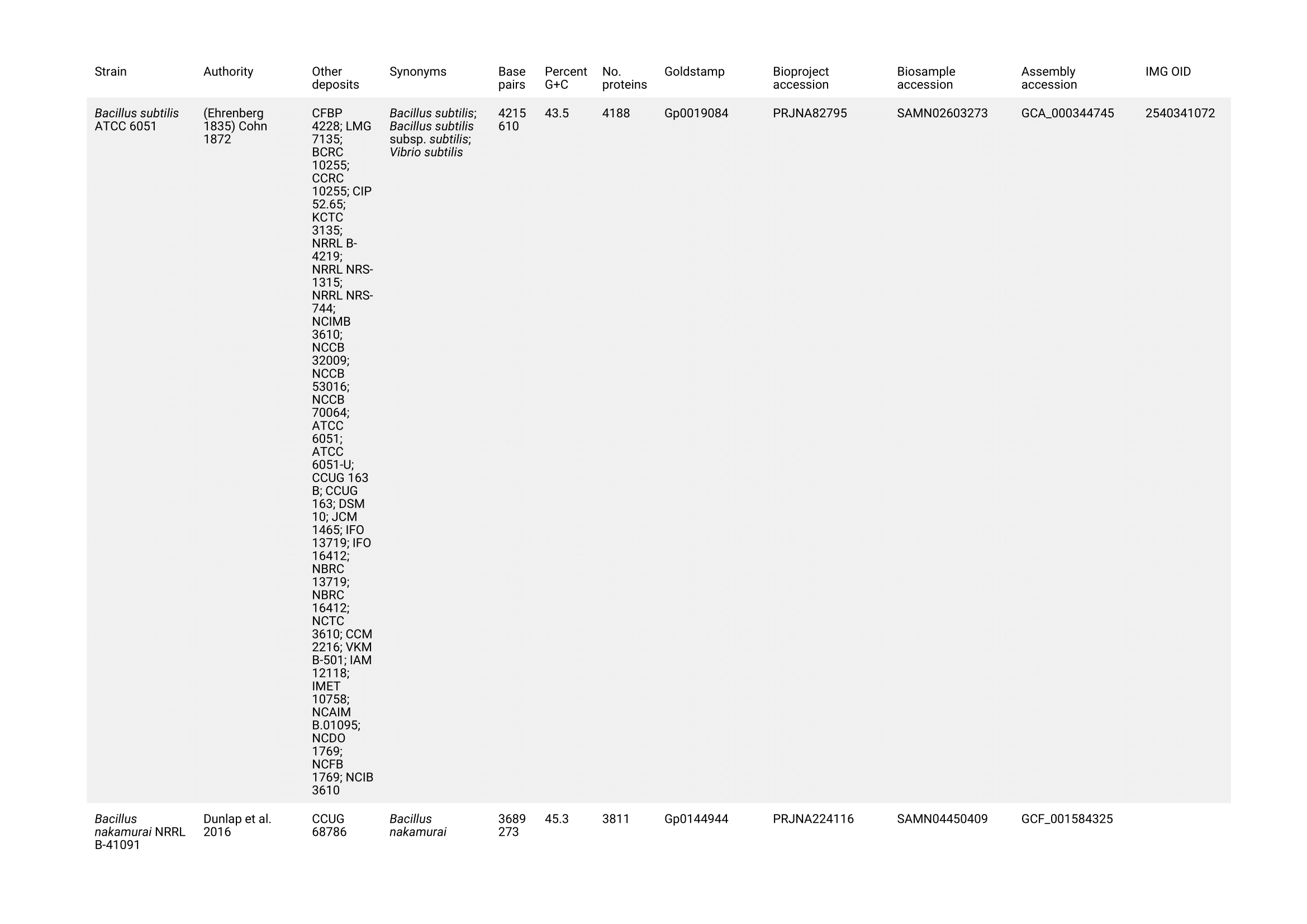

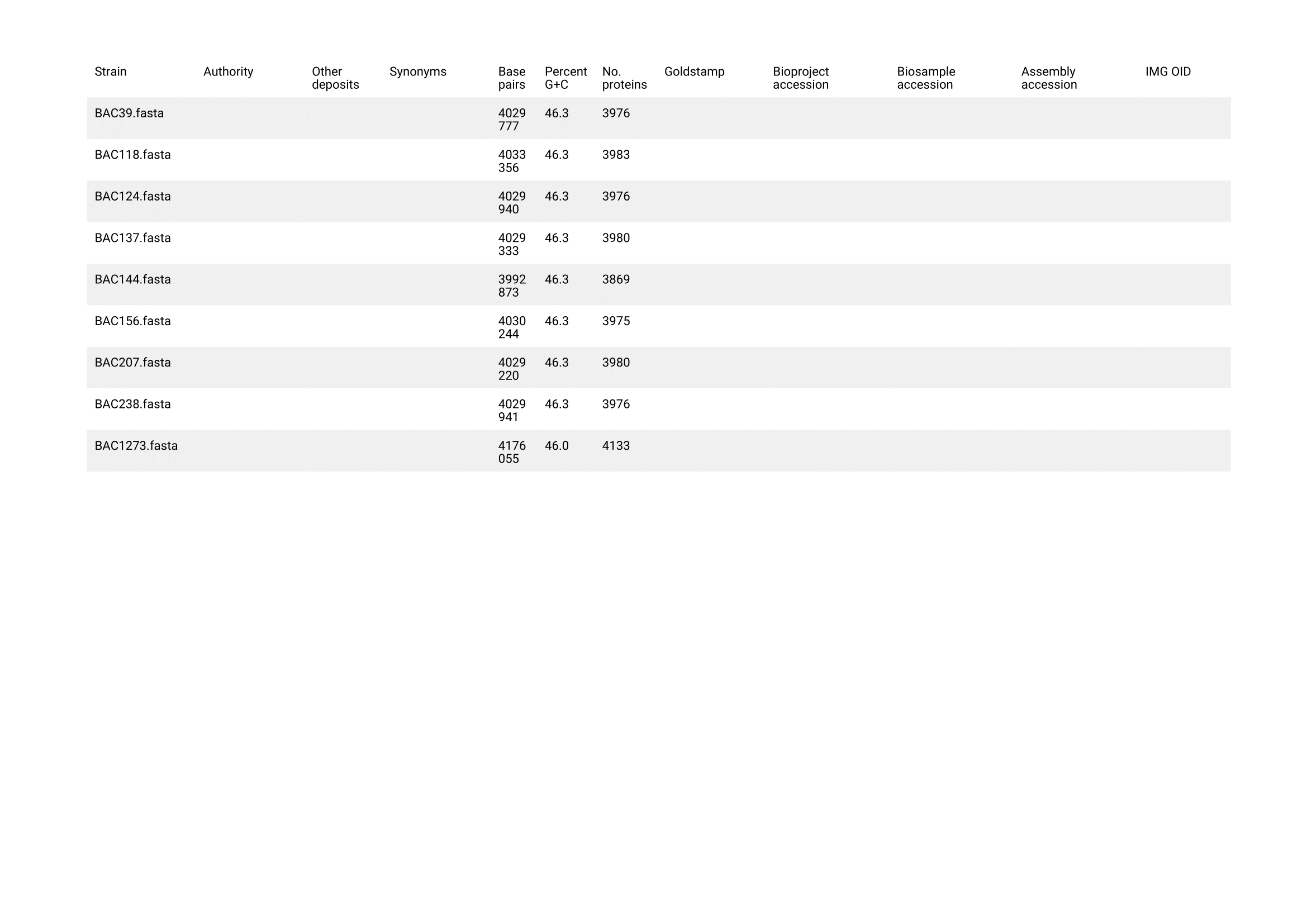

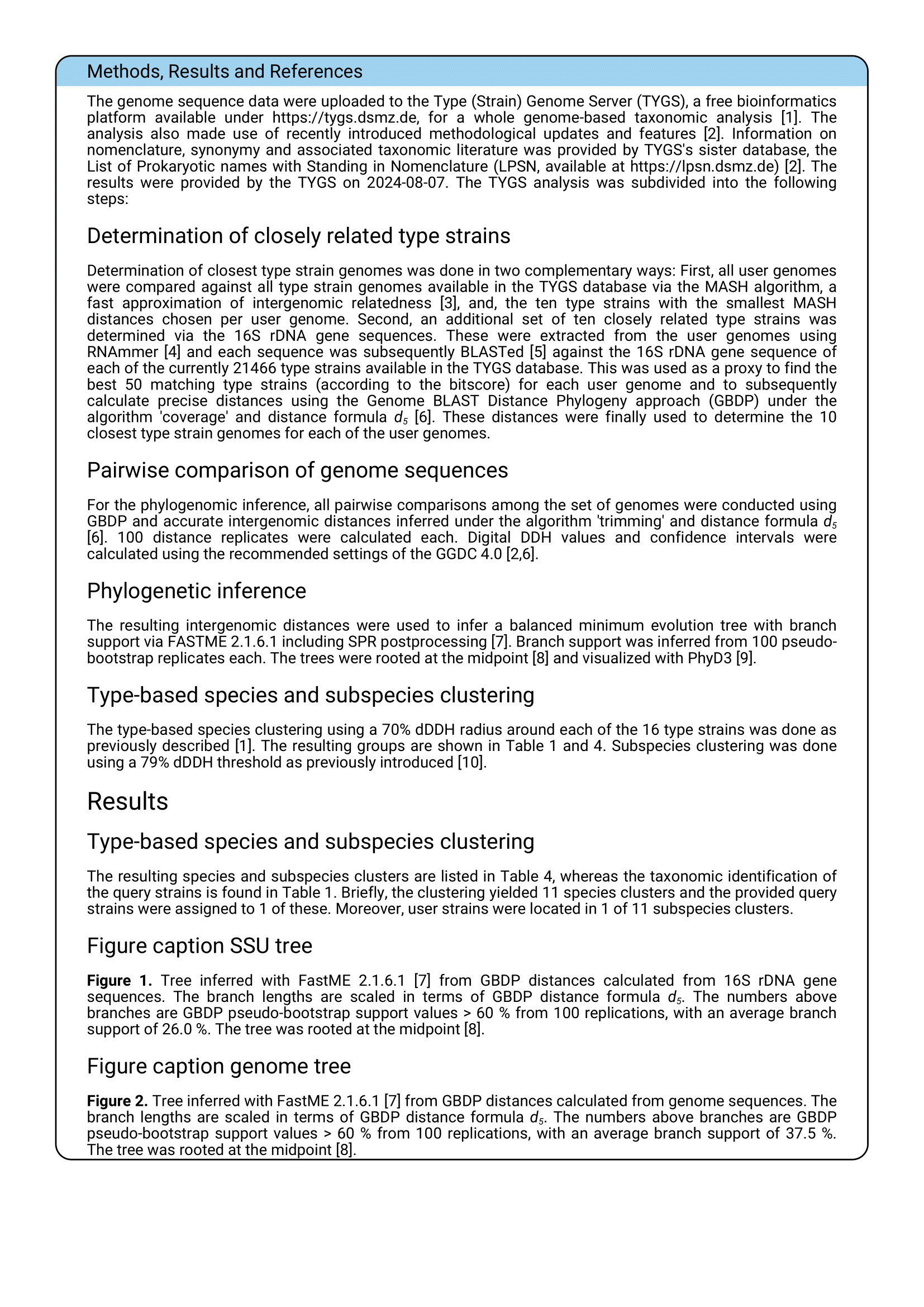

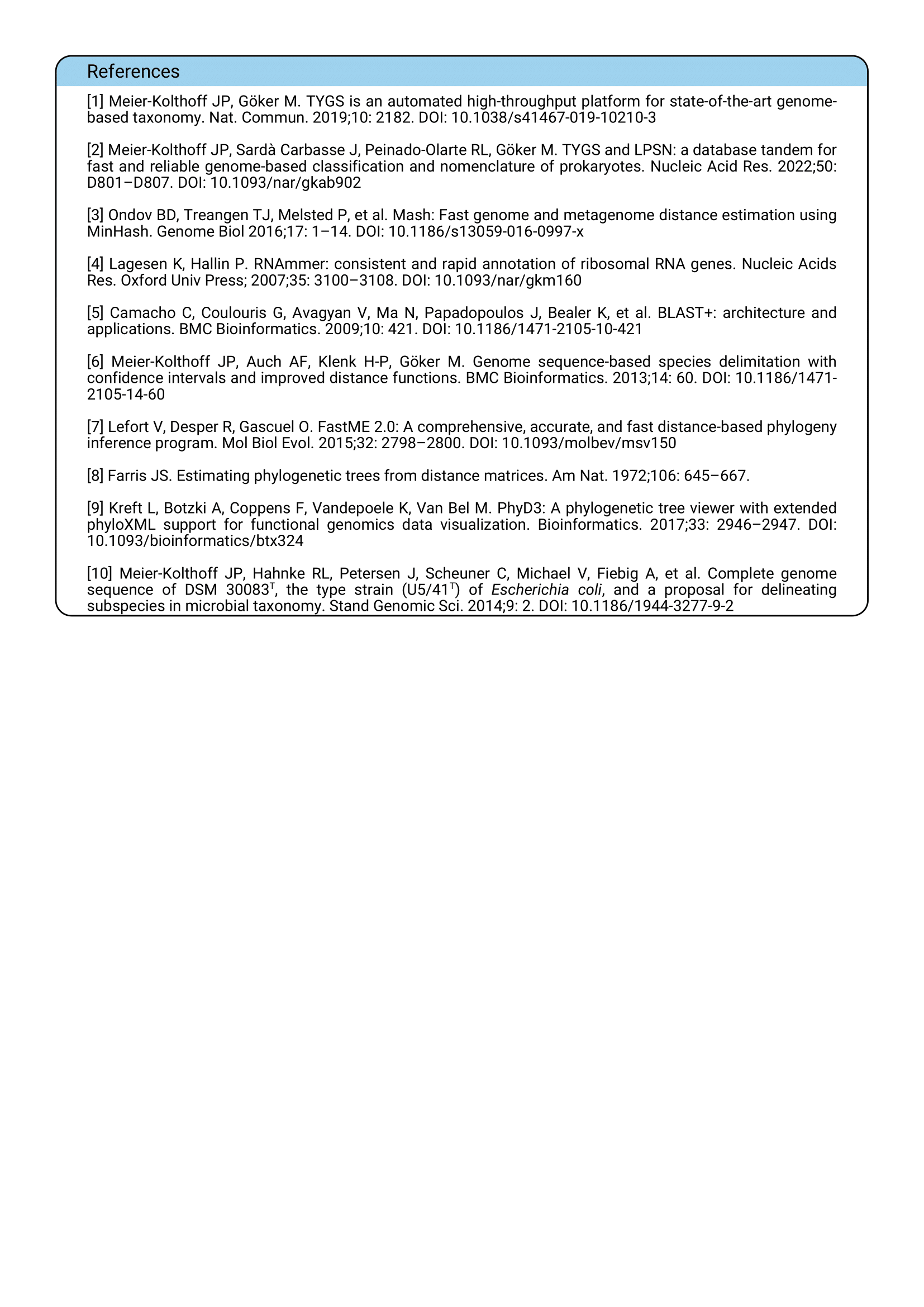


# Supplementary Figures and Table

**2.1 Tables**

**Supplementary Table 1:** Identification of the isolated samples by sequencing, MALDI-TOF and deposit of these genomes in the National Centre for Biotechnology Information (NCBI) database followed by the Bioproject, Biosample and Submission number.

| **Illumina Read ID** | **MALDI-TOF Identification** | **Coverage** | **Name deposited at NCBI** | **Biosample** | **Bioproject** | **Submission** |
| --- | --- | --- | --- | --- | --- | --- |
| VSF981 | BAC118 | 282 | Bv Uniclon 01 | SAMN37733351 | PRJNA1026087 | SUB13890423 |
| VSF985 | BAC144 | 306 | Bv Uniclon 02 | SAMN37734688 | PRJNA1026122 | SUB13907448 |
| VSF1004 | BAC1273 | 233 | Bv Uniclon 03 | SAMN37734915 | PRJNA1026128 | SUB13907769 |
| VSF978 | BAC39 | 274 | Bv Uniclon 04 | SAMN43408009 | PRJNA1154234 | SUB14711318 |
| VSF983 | BAC124 | 278 | Bv Uniclon 05 | SAMN43408010 | PRJNA1154234 | SUB14711318 |
| VSF984 | BAC137 | 314 | Bv Uniclon 06 | SAMN43408011 | PRJNA1154234 | SUB14711318 |
| VSF986 | BAC156 | 226 | Bv Uniclon 07 | SAMN43408012 | PRJNA1154234 | SUB14711318 |
| VSF988 | BAC207 | 323 | Bv Uniclon 08 | SAMN43408013 | PRJNA1154234 | SUB14711318 |
| VSF991 | BAC238 | 282 | Bv Uniclon 09 | SAMN43408014 | PRJNA1154234 | SUB14711318 |

**Supplementary Table 2:** Genomes samples of genus Bacillus used for phylogenomic analyses.

| **Genbank Access** | **Organism** | **Geographic location of isolation** |
| --- | --- | --- |
| GCF_009183655.1 | *Bacillus aerolatus* | China |
| GCF_001884185.1 | *Bacillus albus* | China |
| GCF_002019795.1 | *Bacillus alkalicellulosilyticus* | China |
| GCF_002797415.1 | *Bacillus alkalisoli* | China |
| GCF_000691145.1 | *Bacillus altitudinis* | India |
| GCF_000196735.1 | *Bacillus amyloliquefaciens* | Germany |
| GCF_001244735.1 | *Bacillus andreraoultii* | France |
| GCF_000742895.1 | *Bacillus anthracis* | Unknown |
| GCF_019915265.1 | *Bacillus aquiflavi* | China |
| GCF_001584335.1 | *Bacillus atrophaeus* | United States |
| GCF_001307105.1 | *Bacillus australimaris* | China |
| GCF_001591605.1 | *Bacillus badius* | Japan |
| GCF_014207535.1 | *Bacillus benzoevorans* | United States |
| GCF_000299035.1 | *Bacillus bingmayongensis* | China |
| GCF_004124315.2 | *Bacillus cabrialesii* | Mexico |
| GCF_000007825.1 | *Bacillus cereus* | France |
| GCF_000746925.1 | *Bacillus clarus* | Papua-Nova Guiné |
| GCF_000171615.1 | *Bacillus coahuilensis* | Mexico |
| GCF_000017425.1 | *Bacillus cytotoxicus* | United States |
| GCF_900156875.1 | *Bacillus dakarensis* | France |
| GCF_016908875.1 | *Bacillus ectoiniformans* | United States |
| GCF_001456935.1 | *Bacillus enclensis* | India |
| GCF_012955605.1 | *Bacillus fonticola* | China |
| GCF_002746455.1 | *Bacillus fungorum* | China |
| GCF_001590835.1 | *Bacillus gaemokensis* | South Korea |
| GCF_001042475.2 | *Bacillus glycinifermentans* | South Korea |
| GCF_001278705.1 | *Bacillus gobiensis* | China |
| GCF_001517105.1 | *Bacillus halotolerans* | Morocco |
| GCF_001969855.1 | *Bacillus haynesii* | Israel |
| GCF_018332515.1 | *Bacillus hominis* | Japan |
| GCF_000332645.1 | *Bacillus inaquosorum* | South Korea |
| GCF_022809095.1 | *Bacillus kexueae* | China |
| GCF_001375515.1 | *Bacillus kwashiorkori* | France |
| GCF_000011645.1 | *Bacillus licheniformis* | Denmark |
| GCF_001884105.1 | *Bacillus luti* | China |
| GCF_000712595.1 | *Bacillus manliponensis* | China |
| GCF_902168435.1 | *Bacillus marasmi* | France |
| GCF_001644195.2 | *Bacillus marinisedimentorum* | China |
| GCF_900098925.1 | *Bacillus massiliglaciei* | France |
| GCF_000380245.2 | *Bacillus massiliogorillae* | Unknown |
| GCF_900117315.1 | *Bacillus massilionigeriensis* | France |
| GCF_900111815.1 | *Bacillus mediterraneensis* | France |
| GCF_011008845.1 | *Bacillus mesophilus* | China |
| GCF_000262755.1 | *Bacillus methanolicus* | Unknown |
| GCF_001884045.1 | *Bacillus mobilis* | China |
| GCF_000245335.1 | *Bacillus mojavensis* | United States |
| GCF_000832605.1 | *Bacillus mycoides* | United States |
| GCF_001584325.1 | *Bacillus nakamurai* | Argentina |
| GCF_000612805.1 | *Bacillus ndiopicus* | Unknown |
| GCF_001375535.1 | *Bacillus niameyensis* | France |
| GCF_001884135.1 | *Bacillus nitratireducens* | China |
| GCF_014836955.1 | *Bacillus norwichensis* | United Kingdom |
| GCF_900207585.1 | *Bacillus oleivorans* | United States |
| GCF_016908495.1 | *Bacillus pakistanensis* | United States |
| GCF_001042485.2 | *Bacillus paralicheniformis* | South Korea |
| GCF_001884235.1 | *Bacillus paramycoides* | China |
| GCF_001883995.1 | *Bacillus paranthracis* | China |
| GCF_001884065.1 | *Bacillus proteolyticus* | China |
| GCF_000161455.1 | *Bacillus pseudomycoides* | United States |
| GCF_900186955.1 | *Bacillus pumilus* | Unknown |
| GCF_016464375.1 | *Bacillus renqingensis* | China |
| GCF_917563915.1 | *Bacillus rhizoplanae* | France |
| GCF_000691165.1 | *Bacillus safensis* | United States |
| GCF_003581585.1 | *Bacillus salacetis* | Thailand |
| GCF_004358205.1 | *Bacillus salipaludis* | China |
| GCF_018332475.1 | *Bacillus sanguinis* | Japan |
| GCF_020519665.1 | *Bacillus shivajii* | China |
| GCF_000262045.1 | *Bacillus siamensis* | South Korea |
| GCF_900156865.1 | *Bacillus sinesaloumensis* | France |
| GCF_001050115.1 | *Bacillus smithii* | United States |
| GCF_001742425.1 | *Bacillus solimangrovi* | South Korea |
| GCF_002797395.1 | *Bacillus solitudinis* | China |
| GCF_001592005.1 | *Bacillus sonorensis* | Japan |
| GCF_017939705.1 | *Bacillus suaedae* | China |
| GCF_016890225.1 | *Bacillus suaedaesalsae* | China |
| GCF_000227465.1 | *Bacillus spizizenii* | United States |
| GCF_000009045.1 | *Bacillus subtilis* | Unknown |
| GCF_001969815.1 | *Bacillus swezeyi* | Israel |
| GCF_003318295.1 | *Bacillus taeanensis* | China |
| GCF_000507145.1 | *Bacillus tequilensis* | South Korea |
| GCF_001243895.1 | *Bacillus testis* | France |
| GCF_002119445.1 | *Bacillus thuringiensis* | South Korea |
| GCF_016908565.1 | *Bacillus tianshenii* | United States |
| GCF_000285535.1 | *Bacillus timonensis* | France |
| GCF_000496285.1 | *Bacillus toyonensis* | Japan |
| GCF_001884035.1 | *Bacillus tropicus* | China |
| GCF_004116955.1 | *Bacillus vallismortis* | United States |
| GCF_001461825.1 | *Bacillus velezensis* | Spain |
| GCF_001889165.1 | *Bacillus weihaiensis* | China |
| GCF_001583695.1 | *Bacillus wiedmannii* | United States |
| GCF_001685015.1 | *Bacillus wudalianchiensis* | China |
| GCF_000300535.1 | *Bacillus xiamenensis* | China |
| GCF_002797355.1 | *Bacillus xiapuensis* | China |
| GCF_000715205.1 | *Bacillus zhangzhouensis* | China |
| GCF_001461825.1 | *Bacillus velezensis* | Spain |
| GCF_000015785.2 | *Bacillus amyloliquefaciens subsp. plantarum* | Germany |
| GCF_001045685.1 | *Pseudomonas aeruginosa* |  |

**Supplementary Table 3:** Genome features of isolates identified as *Bacillus velezensis.*

| **ID** | **NAME** | **GUNC** | **CheckM2 Completeness** | **CheckM2 Contamination** | **Coverage** | **Barrnap 5S** | **Barrnap 16S** | **Barrnap 23S** | **CDS** | **rRNA** | **tRNA** |
| --- | --- | --- | --- | --- | --- | --- | --- | --- | --- | --- | --- |
| VSF978 | BAC39 | TRUE | 100.0 | 0.07 | 274 | Complete | Partial | Complete | *3953* | 3 | 76 |
| VSF981 | BAC118 | TRUE | 100.0 | 0.07 | 282 | Complete | Partial | Complete | *3956* | 3 | 82 |
| VSF983 | BAC124 | TRUE | 100.0 | 0.07 | 278 | Complete | Partial | Complete | *3953* | 3 | 76 |
| VSF984 | BAC137 | TRUE | 100.0 | 0.1 | 314 | Complete | Partial | Complete | *3957* | 3 | 76 |
| VSF985 | BAC144 | TRUE | 100.0 | 0.22 | 306 | Complete | Complete | Partial | *3840* | 5 | 82 |
| VSF986 | BAC156 | TRUE | 100.0 | 0.07 | 226 | Complete | Partial | Complete | *3954* | 3 | 76 |
| VSF988 | BAC207 | TRUE | 100.0 | 0.07 | 323 | Complete | Partial | Complete | *3952* | 3 | 76 |
| VSF991 | BAC238 | TRUE | 100.0 | 0.07 | 282 | Complete | Partial | Complete | *3954* | 3 | 76 |
| VSF1004 | BAC1273 | TRUE | 100.0 | 0.26 | 233 | Complete | Complete | Complete | *4125* | 3 | 84 |

**Supplementary Table 4:** Analysis of Housekeeping Genes and MLST Profiles of *Bacillus velezensis* Isolates

| BAC118.fasta | bsubtilis | new_ST | glpF(34) | ilvD(33) | pta(46) | purH(39) | pycA(47) | rpoD(31) | tpiA(60) |
| --- | --- | --- | --- | --- | --- | --- | --- | --- | --- |
| BAC124.fasta | bsubtilis | new_ST | glpF(34) | ilvD(33) | pta(46) | purH(39) | pycA(47) | rpoD(31) | tpiA(60) |
| BAC1273.fasta | bsubtilis | new_ST | glpF(14) | ilvD(13) | pta(29) | purH(33) | pycA(15) | rpoD(35) | tpiA(28) |
| BAC137.fasta | bsubtilis | new_ST | glpF(34) | ilvD(33) | pta(46) | purH(39) | pycA(47) | rpoD(31) | tpiA(60) |
| BAC144.fasta | bsubtilis | new_alleles | glpF(35) | ilvD(34) | pta(15) | purH(~103) | pycA(80) | rpoD(13) | tpiA(~32) |
| BAC156.fasta | bsubtilis | new_ST | glpF(34) | ilvD(33) | pta(46) | purH(39) | pycA(47) | rpoD(31) | tpiA(60) |
| BAC207.fasta | bsubtilis | new_ST | glpF(34) | ilvD(33) | pta(46) | purH(39) | pycA(47) | rpoD(31) | tpiA(60) |
| BAC238.fasta | bsubtilis | new_ST | glpF(34) | ilvD(33) | pta(46) | purH(39) | pycA(47) | rpoD(31) | tpiA(60) |
| BAC39.fasta | bsubtilis | new_ST | glpF(34) | ilvD(33) | pta(46) | purH(39) | pycA(47) | rpoD(31) | tpiA(60) |

**Supplementary Table 5:** Functional diversity of exclusive genes across the three analyzed groups (Subset 7, BAC144, and BAC1273).

| **Group** | **Gene** | **Description** | **COG Category** | **KEGG Orthology** | **PFAMs** |
| --- | --- | --- | --- | --- | --- |
| Exclusively present genes: BAC156, BAC207, BAC238, BAC39, BAC124, BAC137, BAC118 | yqaS | DNA packaging | L | - | HTH_23,Phage_terminase,Terminase_5 |
|  | phoD | Glycerophosphoryl diester phosphodiesterase | C | ko:K01113,ko:K01126 | GDPD,PhoD,PhoD_N |
|  | - | Transcriptional regulator | K | - | WYL |
|  | yobL | Nucleic acid phosphodiester bond hydrolysis | L | ko:K21487 | Endonuclea_NS_2,LXG |
|  | - | Amine dehydrogenase activity | S | - | - |
|  | - | Endonuclease that resolves Holliday junction intermediates in genetic recombination. | L | - | RecU |
|  | - | Phage integrase family | L | - | Phage_int_SAM_1,Phage_int_SAM_4,Phage_int_SAM_5,Phage_integrase |
|  | yhdJ | DNA methylase | L | ko:K00590,ko:K07319 | N6_N4_Mtase |
|  | - | Response regulator | K | - | HNH_3 |
|  | - | Transcriptional regulators containing a DNA-binding HTH domain and an aminotransferase domain (MocR family) and their eukaryotic orthologs | K | ko:K00375 | Aminotran_1_2,GntR |
|  | - | NADH flavin oxidoreductases, Old Yellow Enzyme family | C | ko:K02759 | Oxidored_FMN |
|  | ydhN3 | Phosphotransferase system | G | ko:K02759 | PTS_IIA |
|  | rpoE | DNA-directed RNA polymerase specialized sigma subunit, sigma24 homolog | K | ko:K03088 | Sigma70_r4_2 |
|  |  | Phage capsid family | S | - | Phage_capsid |
|  |  | Helix_turn_helix, mercury resistance | K | - | GyrI-like,MerR_1 |
|  |  | Acetyltransferase | K | ko:K03829 | Acetyltransf_1 |
|  |  | Helix_turn_helix, arabinose operon control protein | K | - | AraC_binding,Cupin_2,HTH_18 |
|  | csbD | CsbD-like | K | - | CsbD |
|  | - | Leucine-zipper of insertion element IS481 | S | ko:K07483 | HTH_23,HTH_28,HTH_29,HTH_Tnp_1 |
| Exclusively present genes BAC144 | licT | Transcriptional antiterminator | K | ko:K03488 | CAT_RBD,PRD |
|  | yncF | Deoxyuridine 5'-triphosphate | F | ko:K01520 | dUTPase |
|  | nrdE | Provides the precursors necessary for DNA synthesis. Catalyzes the biosynthesis of deoxyribonucleotides from the corresponding ribonucleotides | F | ko:K00525 | RNR_N,Ribonuc_red_lgC,Ribonuc_red_lgN |
|  | ddeI | DNA (cytosine-5-)-methyltransferase activity | L | ko:K00558 | DNA_methylase |
| Exclusively present genes BAC1213 | yhdJ | Methyltransferase | AJ | ko:K00571,ko:K00590,ko:K07319 | Methyltransf_11,N6_N4_Mtase,Spermine_synth |
|  | - | Super-infection exclusion protein B | S | - | SieB |
|  | yrkH | Rhodanese Homology Domain | P | - | Lactamase_B,Rhodanese |
|  | - | Transposase IS66 | S | - | Prophage_tail |
|  | xpaC | 5-bromo-4-chloroindolyl phosphate hydrolysis protein | S | - | Halogen_Hydrol |
|  | - | Capsid protein | S | - | Phage_capsid |
|  | mcrA | Restriction endonuclease | L | ko:K07451 | HNH |
|  | dut | Deoxyuridine 5'-triphosphate nucleotidohydrolase | F | ko:K01520 | dUTPase |
|  | exuT | Permeases of the major facilitator superfamily | G | ko:K08191,ko:K08194 | MFS_1 |

**2.2 Figures**

**
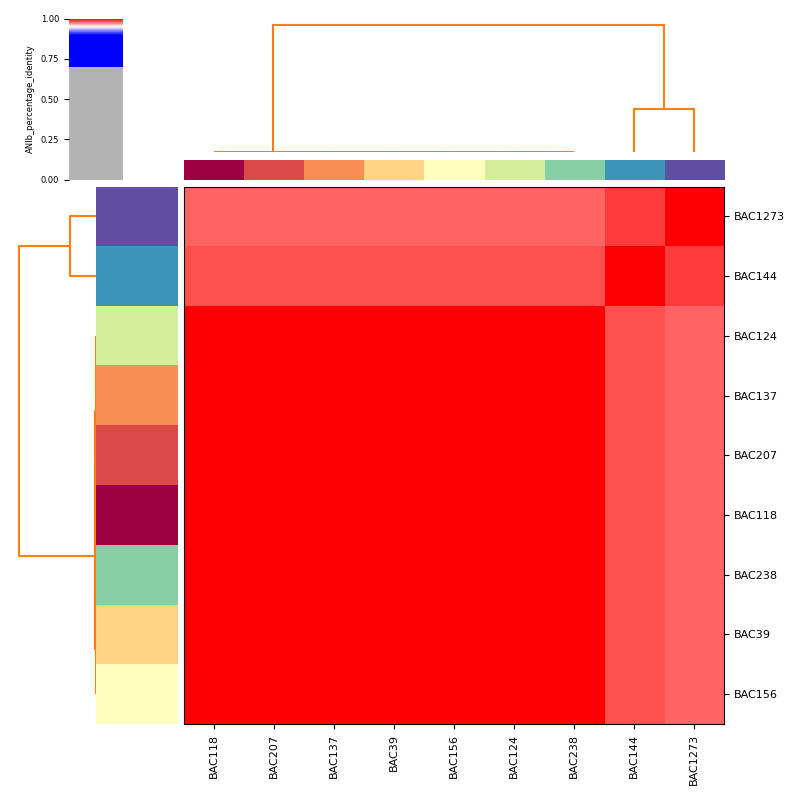
**

**Supplementary Figure 1:** A heatmap of the analysis was carried out to show the similarity percentage between the *Bacillus velezensis* strains isolated in this study demonstrating very high similarity.


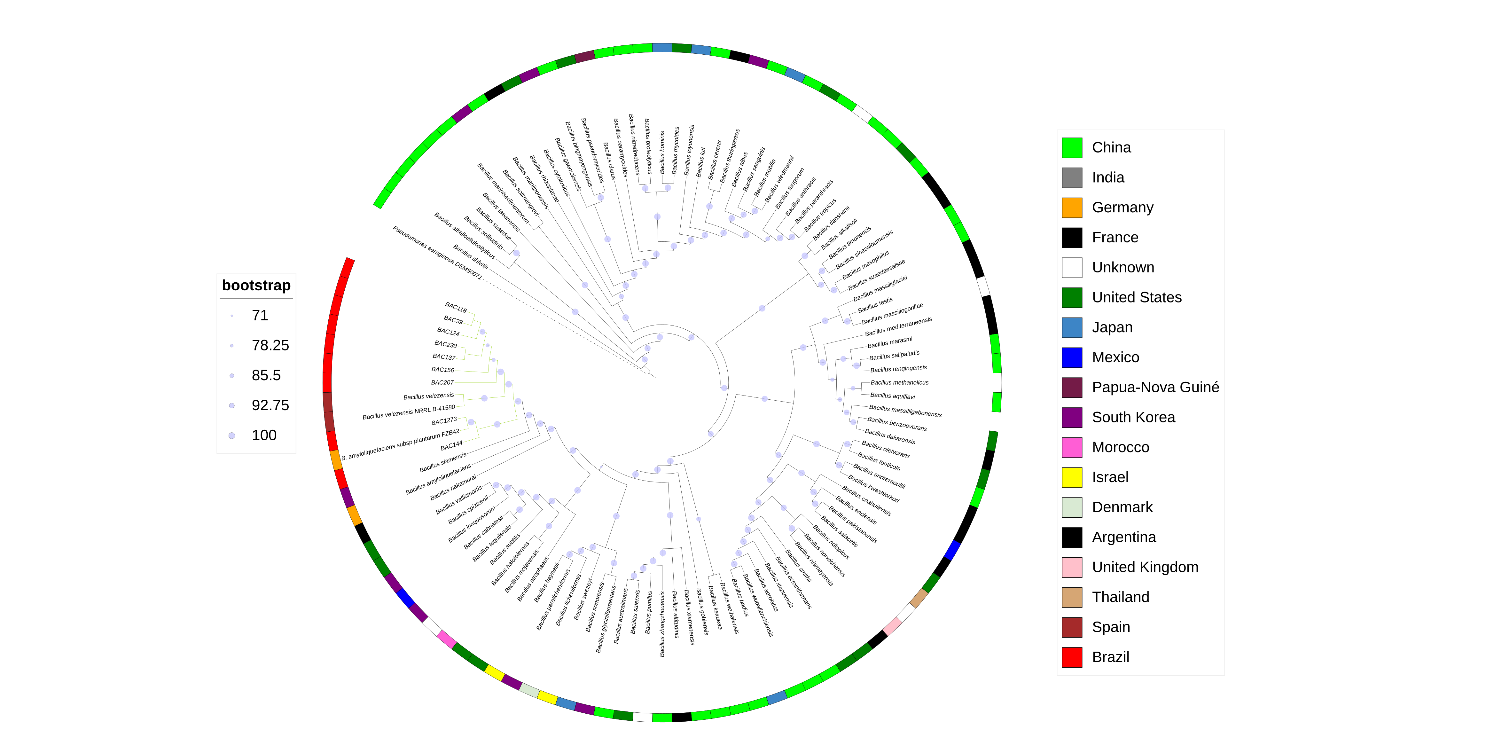


**Supplementary Figure 2:** The phylogenetic tree shows the relationship between all genomes of *B. velezensis strains*. Also, the bootstrap percentage with 1000 bootstraps on the tree branches ranges from 71, the lowest percentage, to 100, the highest.


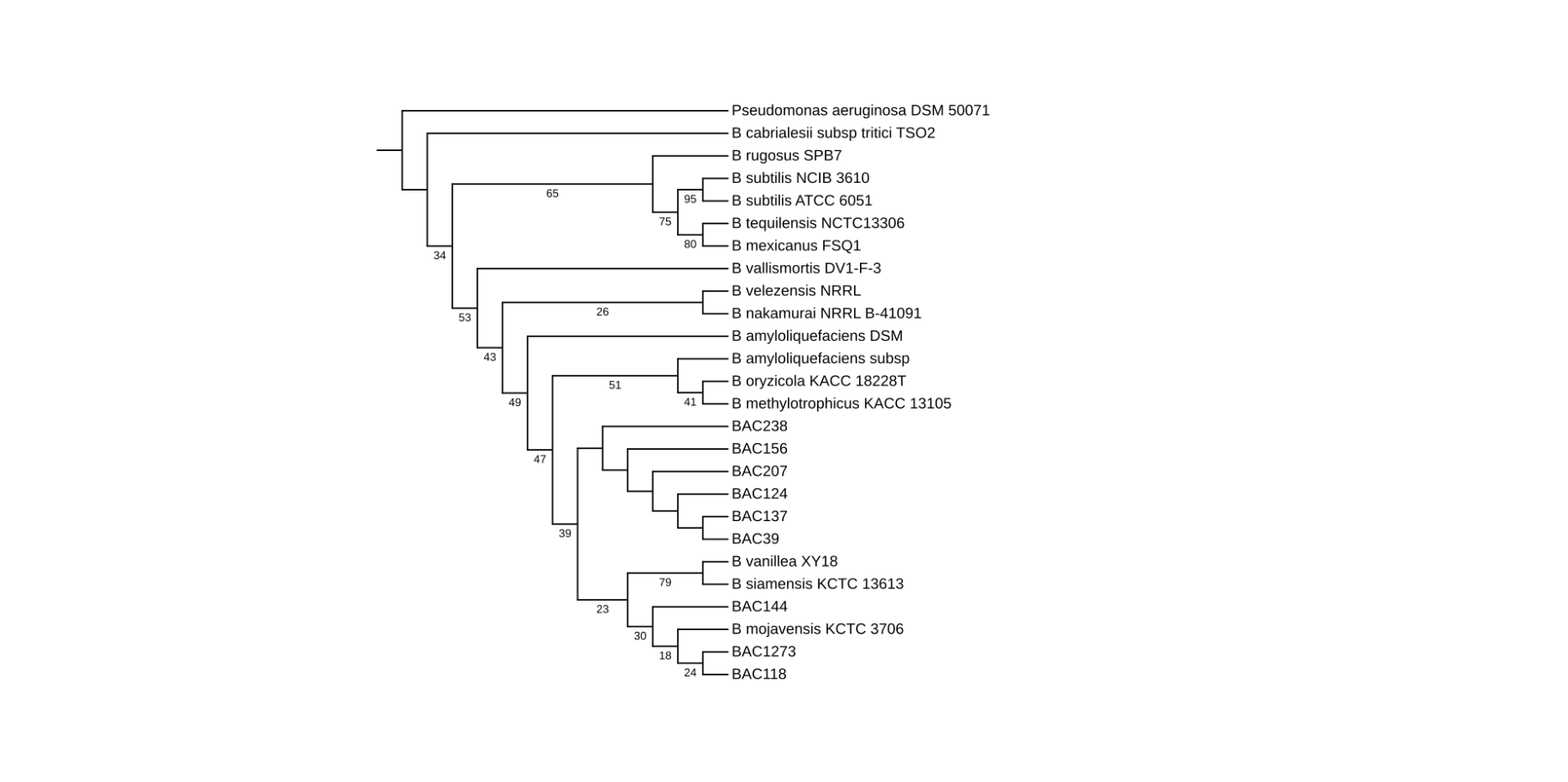


**Supplementary Figure 3:** Phylogenetic tree based on 16S rRNA gene sequences, constructed using the TYGS dataset. The tree was generated using the maximum likelihood method with 1000 bootstrap replicates. The low bootstrap values observed indicate a lack of reliability in the branching positions.


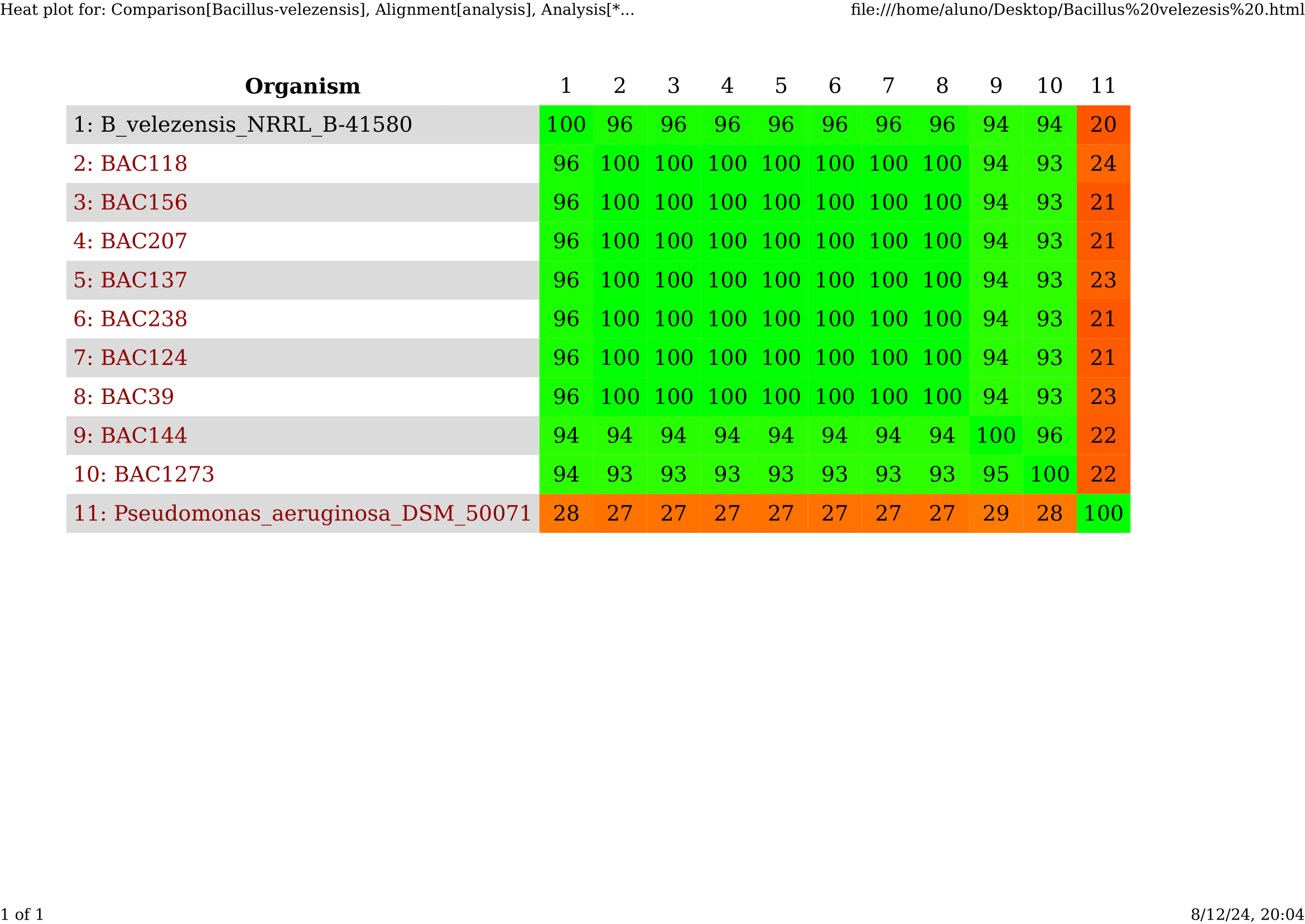


**Supplementary Figure 4:** Heatmap with nine genomes identified as *B. velezensis* in this study and *Bacillus velezensis NRRL B-41580* as representative genome.


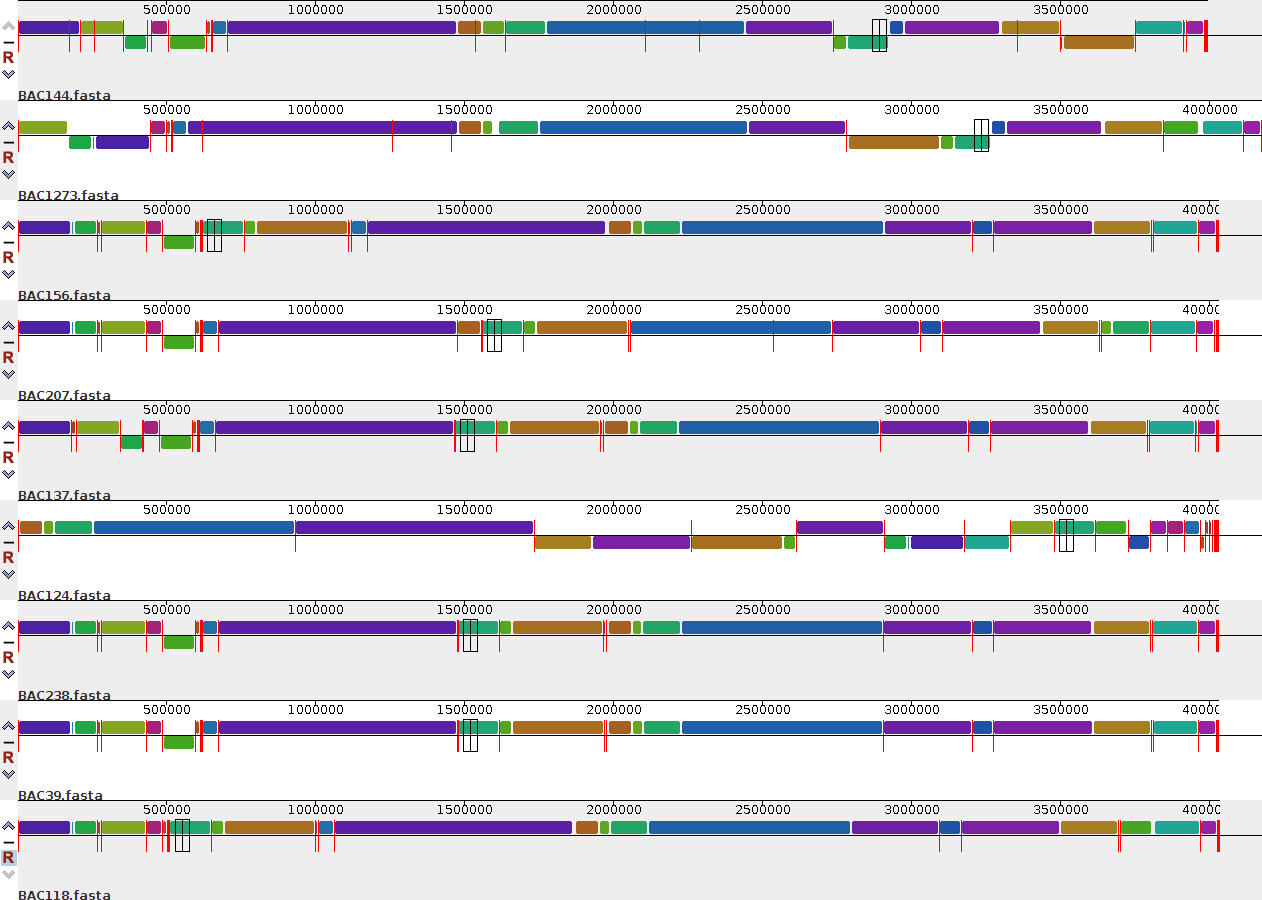


**Supplementary Figure 5:** Comparison between the genomes identified in this study according to synteny between the blocks.
