# Supplementary figures and images for: The research on the identification, taxonomy, and comparative genomics analysis of nine Bacillus velezensis strains significantly contributes to microbiology, genetics, bioinformatics, and biotechnology
Source: Front Microbiol. 2025 Mar 19;16:1544934. doi: 10.3389/fmicb.2025.1544934 (PMC11962042; doi:10.3389/fmicb.2025.1544934)

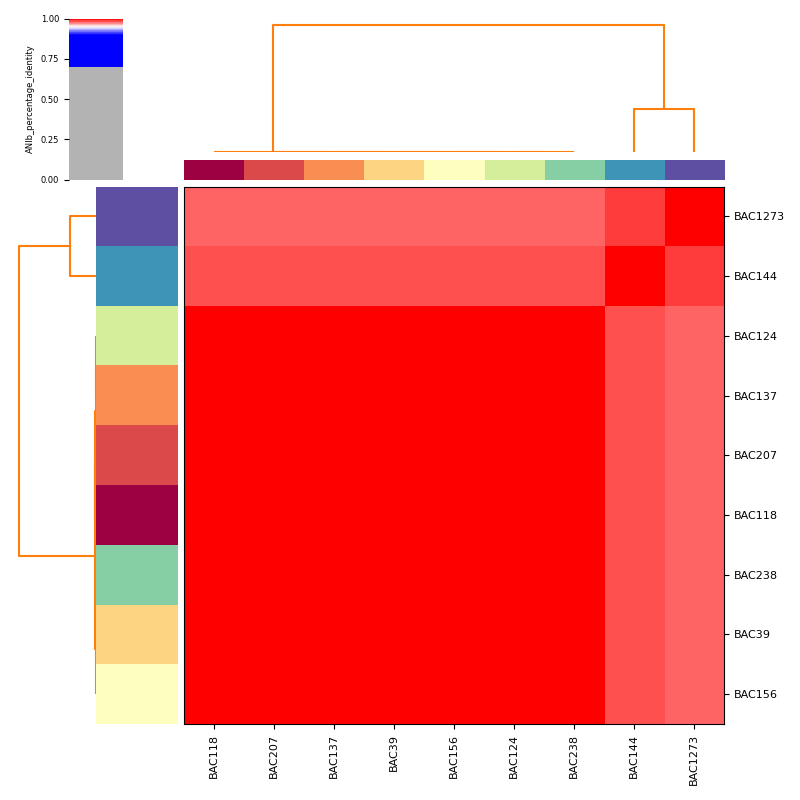

Supplement: Supplementary file 2 [file Image_1.TIFF]

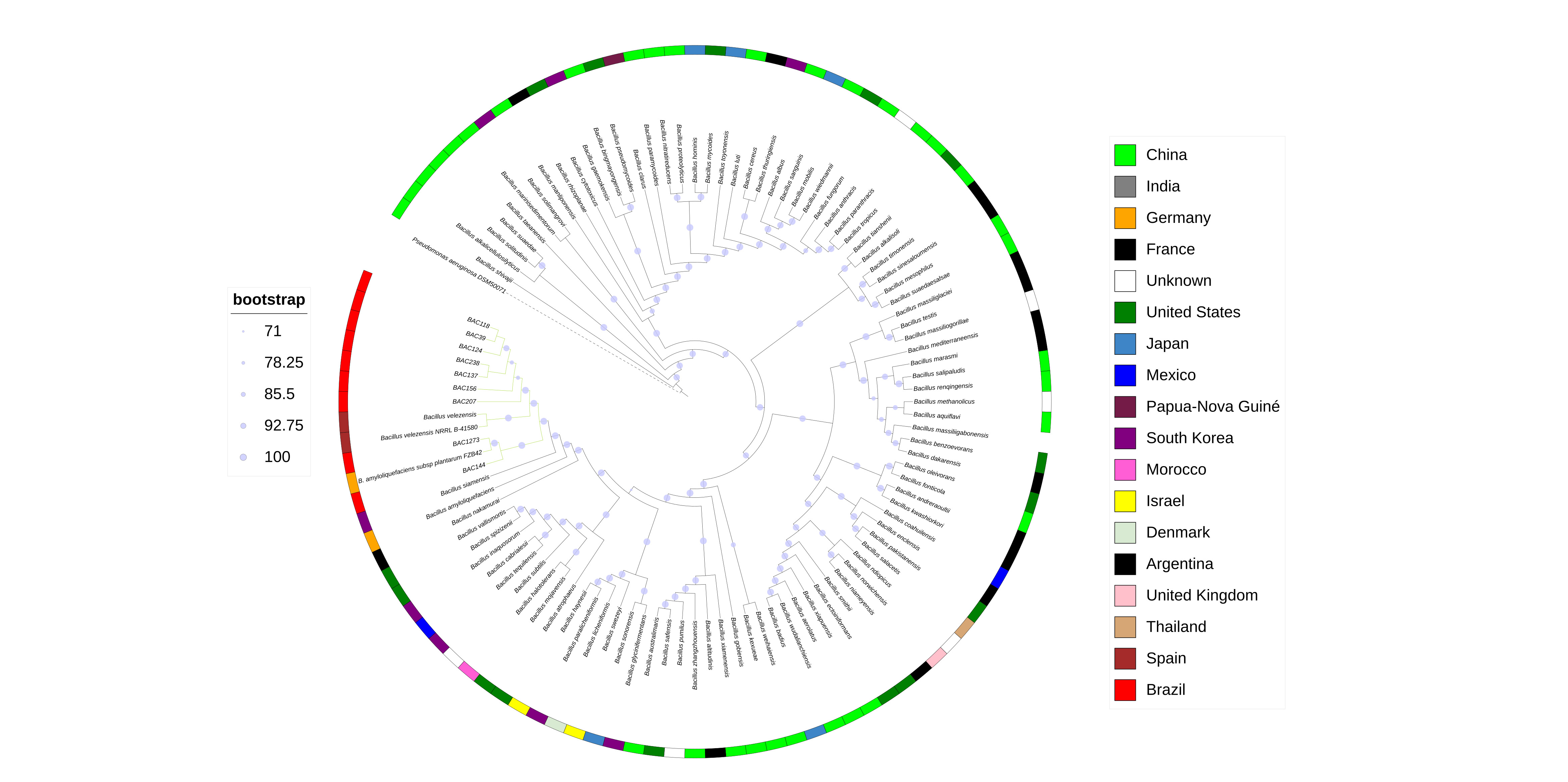

Supplement: Supplementary file 3 [file Image_2.JPEG]

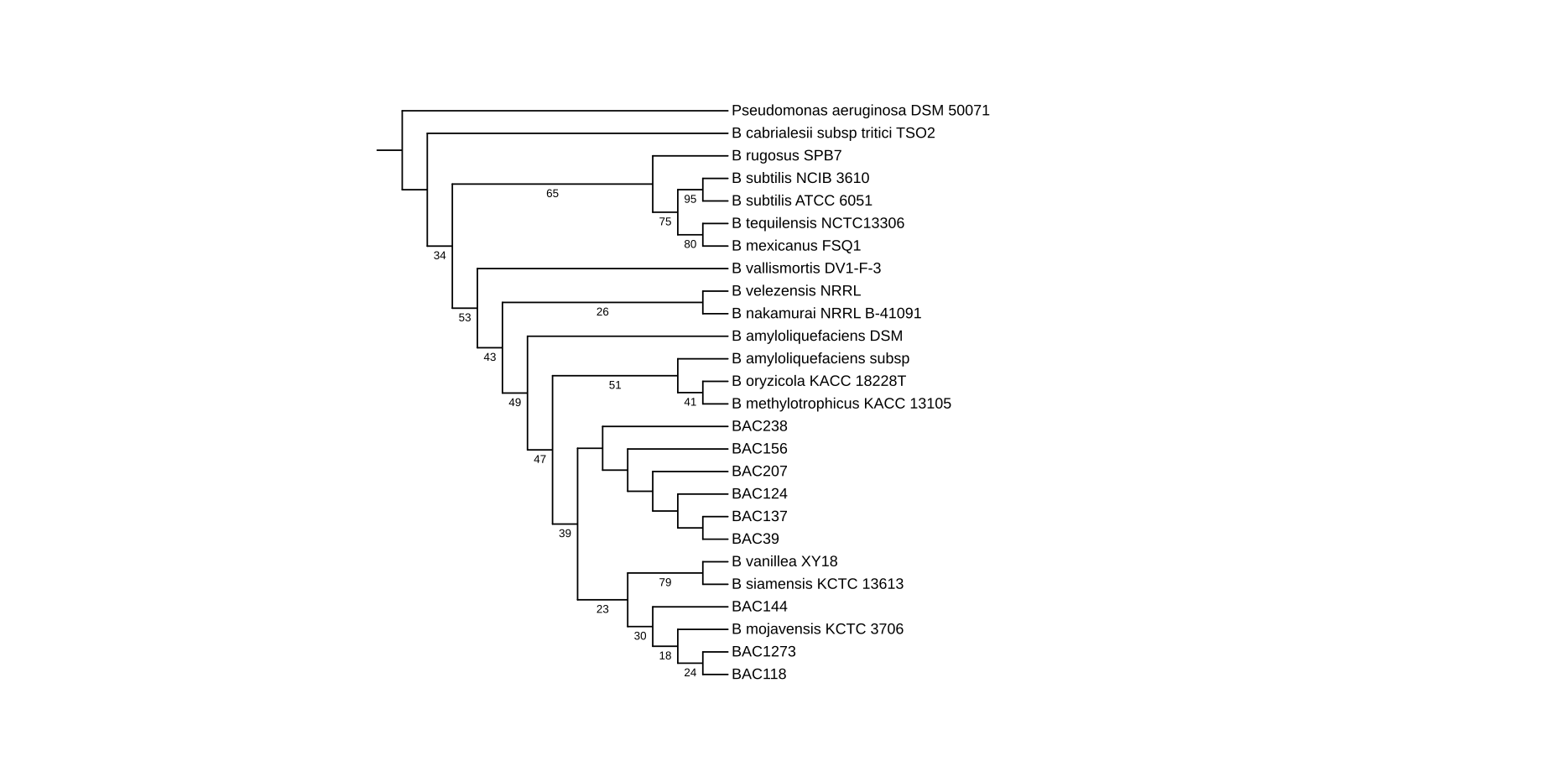

Supplement: Supplementary file 4 [file Image_3.PNG]

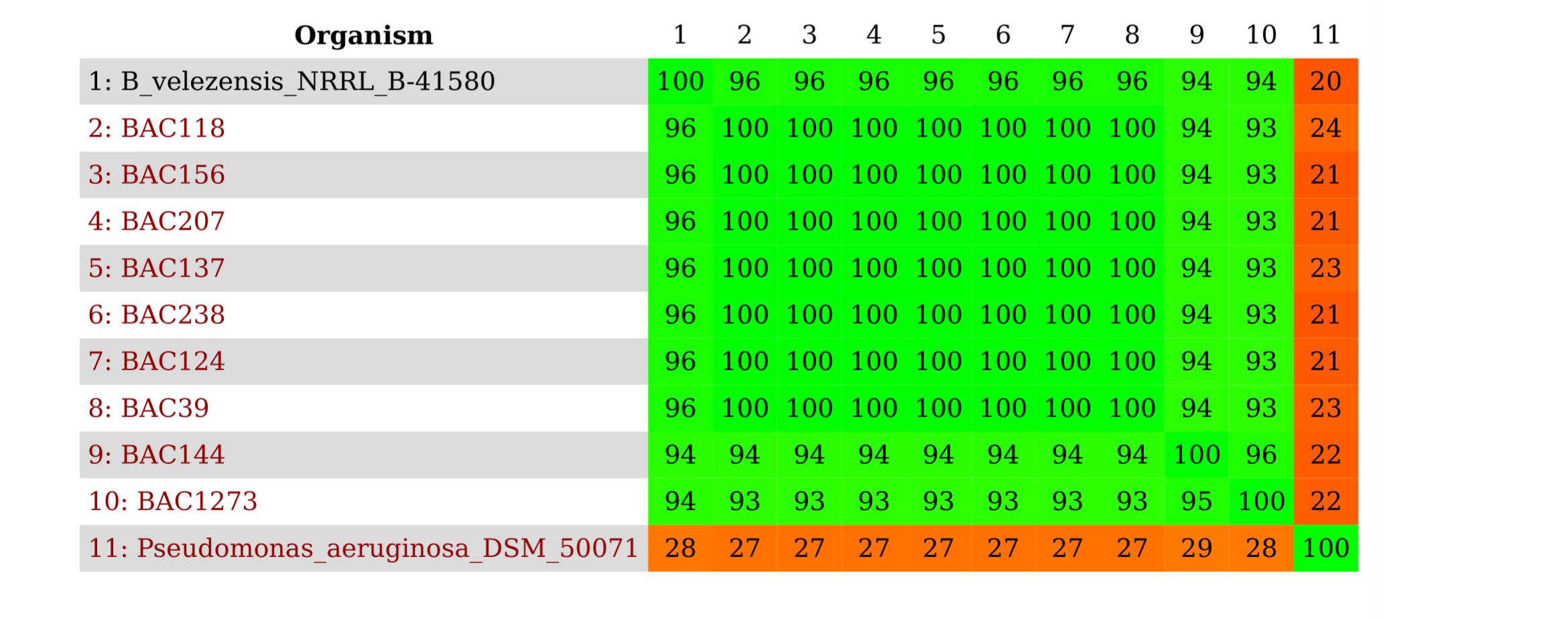

Supplement: Supplementary file 5 [file Image_4.PNG]

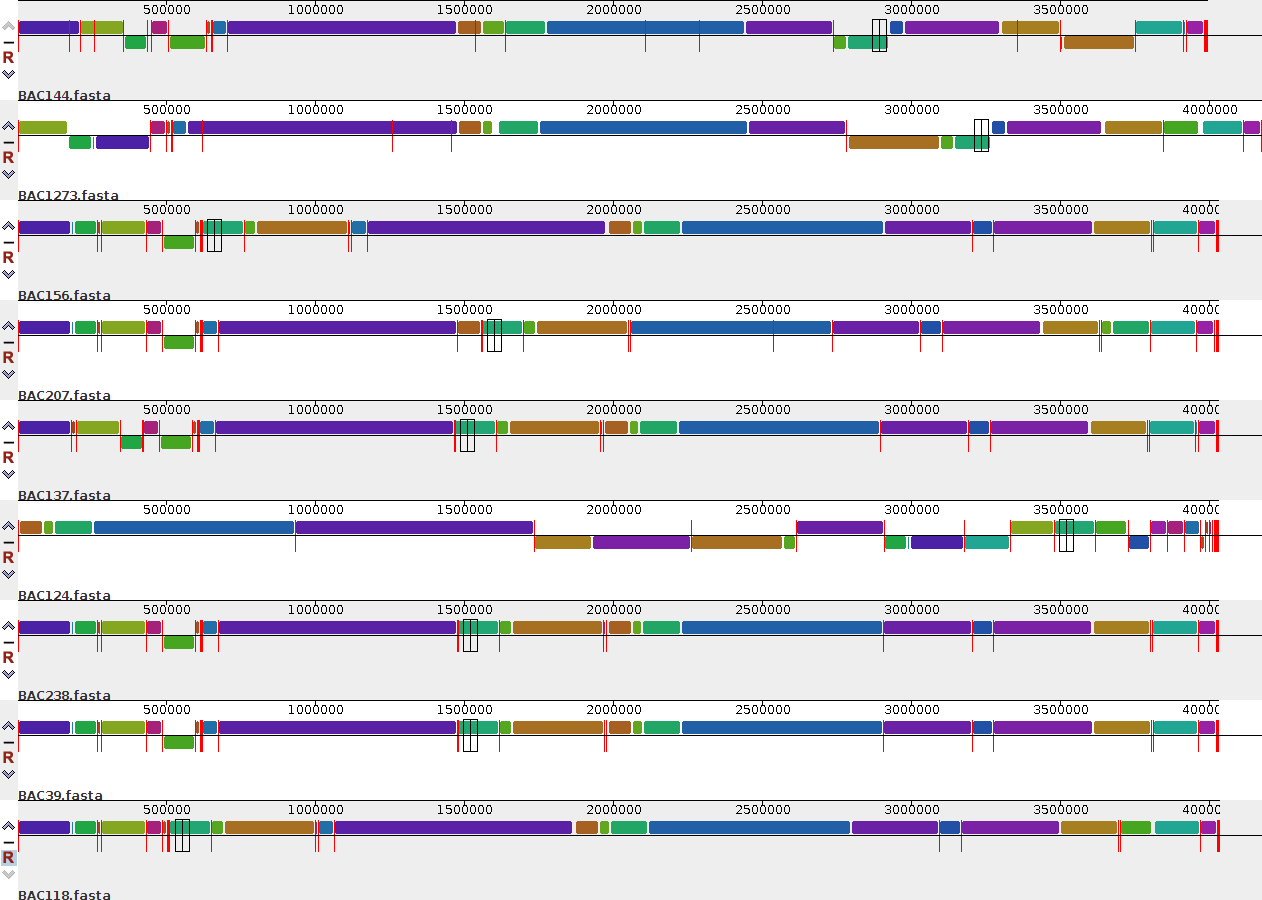

Supplement: Supplementary file 6 [file Image_5.PNG]
